# Magnesium Oxychloride Cement: A Low-Carbon Binder as an Alternative to Portland Cement

**DOI:** 10.3390/ma19091866

**Published:** 2026-05-01

**Authors:** Asad Hanif

**Affiliations:** 1 Civil and Environmental Engineering Department, King Fahd University of Petroleum and Minerals, Dhahran 31261, Saudi Arabia; asad.hanif@kfupm.edu.sa; 2 Interdisciplinary Research Center for Construction and Building Materials, King Fahd University of Petroleum & Minerals, Dhahran 31261, Saudi Arabia

**Keywords:** magnesia-based binder, supplementary cementitious material, fresh properties, mechanical properties, durability, economic and environmental benefit analysis

## Abstract

Magnesium oxychloride cement (MOC), produced from reactive MgO and MgCl_2_, has re-emerged as a promising low-carbon binder due to its rapid setting and high early-age strength. Yet its limited resistance to moisture and immersion remains the principal barrier to broader construction deployment. This review synthesizes the MOC evidence base using a structured approach that combines PRISMA-informed study identification and screening with bibliometric mapping to contextualize research evolution and thematic development. The review follows a structured data extraction of mix design, curing conditions, characterization methods, and performance outcomes. The synthesis confirms that MOC performance is strongly system-dependent. MgO reactivity, MgCl_2_ concentration, mixture ratios, and curing regime govern hydration products, microstructure, and durability, accounting for the apparent variation across studies. Comparative assessment shows that improvements in water resistance are most consistently reported for phosphate-based modification, SCM incorporation, and polymer/hybrid strategies. However, benefits are frequently accompanied by trade-offs in workability, setting, strength development, and cost, and reinforcement compatibility and corrosion risk remain insufficiently resolved for structural applications. The review highlights gaps in reporting and durability testing that currently limit cross-study comparability and translation, and it consolidates priority research directions toward standardized protocols, mechanism-based durability design, scale-up validation, and robust sustainability assessment.

## 1. Introduction

Magnesium oxychloride cement (MOC) is a distinct cementitious binder compared with Ordinary Portland Cement (OPC), as it is primarily produced from reactive magnesium oxide (MgO) and magnesium chloride (MgCl_2_) as the main reactants. MOC was discovered and patented by Stanislas Sorel in the mid-1800s [1]. In simplified terms, MOC formation begins with the conversion of MgO into magnesium hydroxide (Mg (OH)_2_) [2], which subsequently reacts with MgCl_2_ to form magnesium oxychloride hydrate phases [3,4,5]. The overall reaction mechanism is governed by the ternary system MgO-MgCl_2_-H_2_O, where water is not only a mixing medium but also an essential reactant controlling dissolution, precipitation, and phase assemblage [6]. Upon mixing MgCl_2_ with water, it dissociates into Mg^2+^ and Cl^−^, while MgO hydrates and releases Mg^2+^ and OH^−^; the interaction of these species leads to the precipitation of magnesium oxychloride hydrates, often initially forming a gelatinous matrix that progressively crystallizes [7,8]. The final properties of hardened MOC are therefore strongly governed by the type, morphology, and connectivity of its hydrates and pores, i.e., its microstructure [9,10]. The formation and stability of MOC hydrates depend sensitively on the reactivity of MgO, curing temperature, and mixture molar ratios [11,12,13,14]. It is evidenced and well-documented that the product formed by the reaction between MgO and MgCl_2_ in the presence of water generates four principal crystalline phases [15,16,17,18].

1. Phase 2 (2Mg(OH)_2_ MgCl_2_·4H_2_O).

2. Phase 3 (3Mg(OH)_2_ MgCl_2_·8H_2_O).

3. Phase 5 (5Mg(OH)_2_ MgCl_2_·8H_2_O).

4. Phase 9 (9Mg(OH)_2_ MgCl_2_·5H_2_O).

Among these, phases 3 and 5 are the most stable ones formed at ambient temperatures, unlike the other two phases, which form at temperatures beyond 100 °C [16]. Phases 3 and 5 also tend to exhibit more developed crystallinity and intergrown crystal networks, which contribute to their relative stability and compactness in hardened matrices [19,20]. From a mechanical perspective, maximizing phase 5 is frequently considered desirable because it is commonly associated with higher strength and a denser, more interlocked microstructure [19,20]. However, a key limitation of conventional MOC is its poor water resistance [15], which is often attributed to the instability/hydrolysis of oxychloride hydrates and the leaching of soluble chlorides when exposed to prolonged moisture or immersion processes that can increase porosity and reduce strength over time [21,22]. Importantly, “more phase 5” does not automatically guarantee water durability unless the hydrate assemblage and pore solution chemistry are also controlled to suppress deleterious transformations [15].

Several studies have therefore focused on engineering phase 5 formation kinetics and the resulting pore structure to improve both early strength and water resistance [6,23,24]. One highly relevant example is the use of phase 5 seed crystals, which have been shown to accelerate phase 5 precipitation, refine microstructure, and improve compressive strength; notably, the beneficial effect can be even more pronounced in MOC systems incorporating phosphoric acid (often used as a water-resistance agent), where seeding helps offset slower phase 5 development and promotes a denser matrix [15]. In parallel, phosphoric acid or phosphate modification has been widely reported as an effective route to improve phase development and durability in selected mix designs; for example, phosphoric acid has been shown to shift hydrate assemblage toward phase 5 under otherwise strength-deficient conditions (e.g., high H_2_O/MgCl_2_ ratios), improving strength development through altered hydration products and microstructure [25]. Beyond phosphate-based strategies, supplementary cementitious materials (SCMs), such as fly ash and other aluminosilicate-rich additions, have also demonstrated improved water resistance via microstructural densification and secondary reaction products that reduce permeability and mitigate hydrate degradation [26]. These modification approaches highlight that achieving durable MOC requires a coupled design of hydrate phase assemblage, pore structure, and exposure stability, rather than targeting phase 5 content alone.

MOC also possesses several attractive functional properties. It exhibits excellent fire resistance [26,27] and is known for good bonding performance, resistance to abrasion and certain chemical exposures, and the ability to provide smooth finished surfaces [28]. Owing to its binding efficiency, MOC has been used with various fillers (e.g., sawdust, mineral aggregates, and other particulates), enabling applications in ornamental products, interior paneling, specialty flooring, and insulation-type boards [7]. It is a relatively rapid-setting material compared to OPC [9] and, as such, a candidate for fast repair applications where early strength is beneficial. In the context of sustainability, MOC and related MgO-based cements are frequently discussed as candidates for lower-carbon construction pathways, if production routes, MgO reactivity control, and mixture optimization are carefully managed [29,30,31,32]. Additionally, magnesium-based cement systems have been studied for their potential to reabsorb CO_2_ through carbonation reactions; however, the magnitude and practicality of such uptake depend strongly on exposure conditions and phase assemblage [33,34].

Despite these advantages, practical deployment of MOC in structural applications must address durability and reinforcement-related concerns. MgO-based binders can exhibit lower pore solution pH than OPC systems, which may reduce the natural passivation of conventional steel reinforcement and increase corrosion susceptibility unless mitigation strategies are adopted [31,35]. This has motivated research into mix modification, protective measures, and alternative reinforcement solutions compatible with MgO-based binder chemistries [22,31]. In comparison, OPC, while dominant due to its broad performance record, has well-known drawbacks, including a high CO_2_ footprint [36], susceptibility to certain chemical attacks [37], and brittle cracking tendencies, alongside slow setting and shrinkage-related volume changes in some applications [38]. MOC, on the other hand, has been reported to offer negligible volume change, faster setting, favorable thermal behavior, high fire resistance, and competitive compressive/flexural strength at lower density [39,40]. Consequently, the main research challenge is no longer whether MOC can achieve high strength, but how to deliver strength retention under moisture exposure by stabilizing hydrates and controlling pore structure through scientifically guided mix design and curing strategies [22,41,42].

Although MOC has been reviewed from multiple angles, ranging from early application-oriented summaries [28] to state-of-the-art developments [21] and manufacture–curing–structure–performance linkages [22], the existing review literature still exhibits several limitations that constrain decision-making for engineering deployment. First, many prior reviews are predominantly narrative and therefore provide limited reproducibility in evidence selection, while bibliometric mapping has typically been applied in narrower contexts such as wood–MOC composites [43]. Second, even recent reviews that emphasize opportunities and challenges, ductile composite concepts, and water-resistance improvement [41,42] do not consistently reconcile contradictory findings across studies under harmonized criteria (e.g., mixture ratios, MgO reactivity, curing regimes, and water-resistance test definitions), which makes cross-study comparison difficult. Third, emerging directions, such as multifunctional and lifecycle-aware MOC materials [44], have not yet been fully integrated with construction-focused performance synthesis and practical constraints. In response, the present review contributes a consolidated and critical synthesis of MOC research by integrating systematic evidence selection with research mapping and by translating the fragmented findings into comparative insights on processing–microstructure–property relationships, performance trade-offs, and a focused agenda for future research and implementation [19,20,22].

The objectives of this review are to (i) map the evolution and thematic structure of the MOC research landscape and identify dominant and emerging research clusters; (ii) synthesize the fundamental mechanisms governing hydration, phase assemblage, and microstructure development, and relate them to macroscopic properties; (iii) critically compare manufacturing parameters and curing regimes as key determinants of performance variability; (iv) evaluate and contrast the effectiveness and trade-offs of major property-enhancement strategies including inorganic/organic additives, supplementary cementitious materials, and fiber/polymer modifications particularly for improving water resistance; and (v) distill the collective evidence into practical research needs, standardization priorities, and future directions that can accelerate translation of MOC from laboratory demonstrations to reliable construction applications [22,41,42].

This paper is organized systematically. The introduction is followed by the review methodology and bibliometric workflow used to identify, screen, and map the literature. The subsequent sections discuss the environmental motivation for alternative cementitious systems, followed by the fundamentals of MOC production, hydration, and curing. The manuscript then critically reviews property-enhancement strategies and compares their effects on mechanical performance, microstructure, and water resistance. A dedicated discussion in Section 7 synthesizes cross-study comparisons, resolves key inconsistencies, and derives overarching insights and design-relevant implications. Finally, the conclusions in Section 8 summarize the principal findings and provide a future research perspective for advancing MOC toward durable and scalable construction use.

## 2. Research Methodology and Bibliometric Analysis

Literature synthesis in cementitious materials research encompasses narrative and systematic reviews, as well as bibliometric science mapping. In recent construction materials scholarship, hybrid methodologies integrating PRISMA-guided systematic screening with bibliometric analysis have gained prominence, as they enable transparent study selection while simultaneously revealing macro-scale research patterns, including publication growth, collaboration networks, and thematic evolution [45,46,47]. Such an integrated approach is particularly suitable for magnesium oxychloride cement (MOC), a multidisciplinary research domain encompassing fundamental hydration chemistry, phase assemblage and microstructural development, mechanical performance, durability, and diverse modification strategies [41,42]. Although previous bibliometric investigations have addressed MOC in specific contexts, such as wood–MOC composites [43], a comprehensive construction-oriented synthesis integrating performance, durability, and sustainability perspectives remains limited.

**Figure 1:**
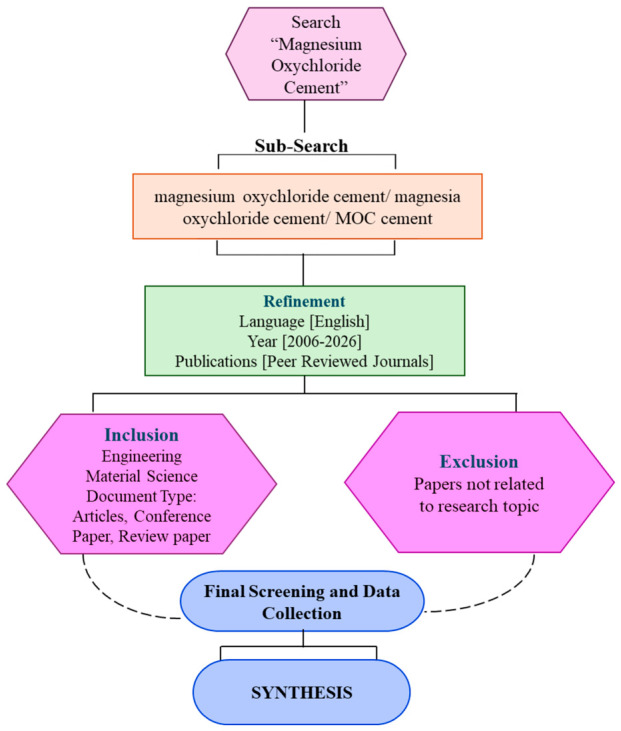
Approach for systematic review.

Accordingly, this study adopts a dual methodological framework comprising a PRISMA-informed systematic review to ensure structured identification, screening, and eligibility assessment of relevant studies, and a bibliometric analysis to map the intellectual structure and developmental trajectory of MOC research. The reporting aligns with the PRISMA 2020 guidelines to promote transparency and reproducibility [41], while protocol formulation was guided by PRISMA-P principles to ensure clarity in scope definition, eligibility criteria, and analytical design [48]. The overview of the approach is shown in Figure 1. The review was designed to address three principal research questions: (a) How has MOC research evolved in terms of publication volume and thematic focus between 2006 and 2026? (b) Which performance domains, including fresh properties, mechanical behavior, durability, and water-resistance enhancement, demonstrate consistent improvements, and under what curing and testing conditions? (c) What methodological inconsistencies in mix design, curing regimes, and testing standards limit cross-study comparability and practical translation? The Scopus database was selected as the primary source for literature retrieval due to its extensive coverage of engineering and materials science journals and its structured bibliographic metadata, which are particularly suitable for bibliometric mapping [49,50]. Compared with other databases such as Web of Science, Scopus provides broader journal coverage within engineering disciplines and facilitates consistent export of citation and keyword datasets for science mapping. Although Web of Science has been used in related bibliometric studies, Scopus was preferred to minimize database bias and ensure comprehensive retrieval of peer-reviewed engineering literature [49,50].

The search was conducted within the “Title–Abstract–Keywords” fields using the core query: “magnesium oxychloride cement” OR “magnesia oxychloride cement” OR “MOC cement” OR “Sorel cement”. To enhance both recall and precision, the query was iteratively refined by incorporating performance- and application-related terms, which is consistent with established practices in bibliometric–systematic reviews of cement-based materials [51]. The timeline was defined as 2006–2026 to capture contemporary research development, and results were restricted to English-language peer-reviewed journal articles and reviews. Study selection followed a sequential screening process comprising automated filtering by publication year and language, followed by title screening, abstract assessment, and full-text evaluation. Studies were included if they investigated MOC as a construction binder or composite matrix (paste, mortar, concrete, or related composites) and reported at least one of the following domains: hydration and phase assemblage, microstructural characterization, fresh-state properties, mechanical performance, durability, modification strategies, or environmental implications relevant to construction applications [41,42]. Studies were excluded if MOC was not the primary binder system.

For each eligible study, structured data extraction captured bibliographic metadata, materials descriptors, curing regimes, specimens, testing protocols, and reported performance outcomes. Performance indicators were grouped into comparable categories, including fresh properties, mechanical performance, durability and water-resistance indicators, and microstructural evidence. Given the heterogeneity in mix designs, curing conditions, and testing methodologies across the MOC literature [41,42], synthesis was conducted as a structured narrative with targeted quantitative comparisons, emphasizing representative performance ranges rather than pooled statistical meta-analysis.

**Figure 2:**
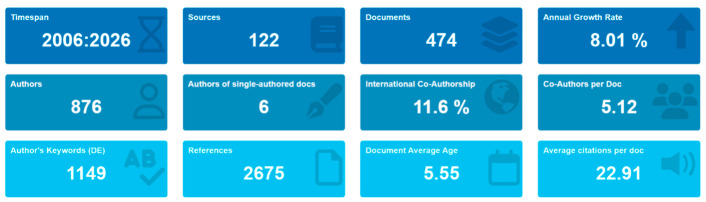
Bibliometric overview of magnesium oxychloride cement (MOC) research (2006–2026).

Bibliometric analysis was conducted in parallel to contextualize the systematic synthesis within the broader knowledge framework of MOC research, following established science mapping methodologies [52,53]. Scopus metadata were exported in CSV format and cleaned to harmonize author names, institutional affiliations, and keywords. Network visualization and co-occurrence mapping were performed using VOS viewer to analyze co-authorship structures and keyword relationships [53], while Bibliometrix/Biblioshiny workflows were employed to generate descriptive indicators and thematic evolution analyses [52]. Figure 2 presents a quantitative bibliometric summary of MOC research published between 2006 and 2026. A total of 474 documents were retrieved from 122 sources, involving 876 contributing authors. The field demonstrates a steady annual growth rate of 8.01%, reflecting sustained and increasing research interest. Collaborative research is evident, with an average of 5.12 co-authors per document and 11.6% international co-authorship. The dataset includes 1149 author keywords and 2675 cited references, indicating thematic diversity and strong scholarly foundations. The average document age of 5.55 years suggests a relatively recent, evolving research domain, while the average citation count of 22.91 per document indicates moderate to high academic impact within the field.

**Figure 3:**
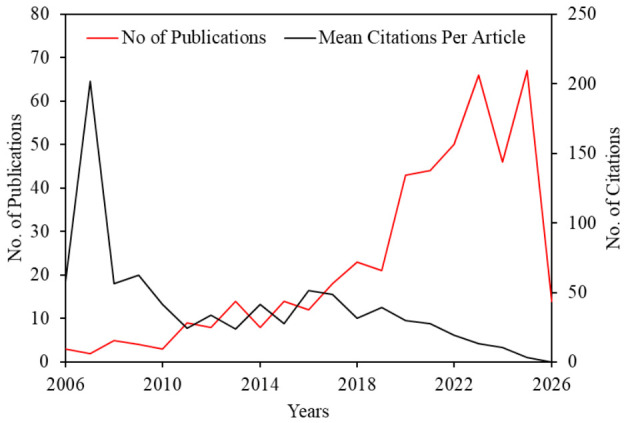
Annual publication output and mean citations per article in MOC research.

Figure 3 illustrates the temporal evolution of MOC research from 2006 to 2026, showing both the annual number of publications and the mean citations per article. The publication trend shows a gradual increase in the early years, followed by a pronounced growth phase after 2018, reaching a peak between 2022 and 2025. In contrast, the mean citations per article show higher values in the earlier years, reflecting citation accumulation over time, followed by a declining trend in recent years due to the shorter citation window of newly published articles. Overall, the figure highlights sustained research expansion alongside maturing citation dynamics within the MOC research domain.

**Figure 4:**
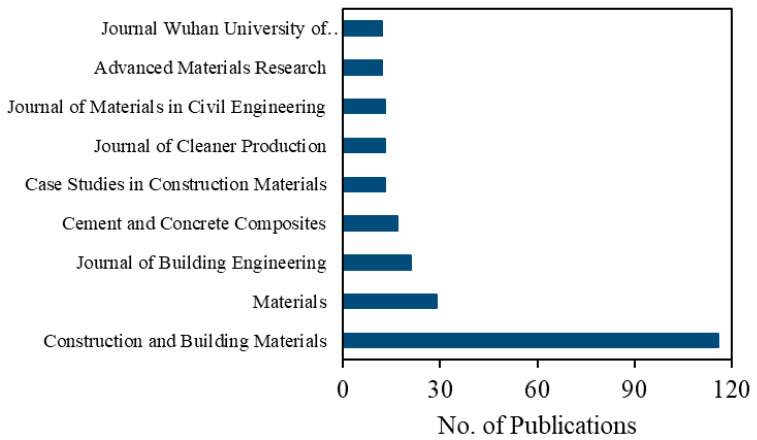
Distribution of publications across leading journals in MOC research.

Figure 4 presents the distribution of magnesium oxychloride cement (MOC) publications across major peer-reviewed journals. Construction and Building Materials clearly dominates the field, accounting for the highest number of publications, indicating its central role in disseminating research on MOC. This is followed by Materials, Journal of Building Engineering, and Cement and Concrete Composites, reflecting strong contributions from materials science and construction-focused journals. Other outlets, including Journal of Cleaner Production and Case Studies in Construction Materials, demonstrate growing interdisciplinary engagement, particularly in sustainability and applied construction research. The distribution highlights that MOC research is primarily concentrated within established construction materials and civil engineering journals.

**Figure 5:**
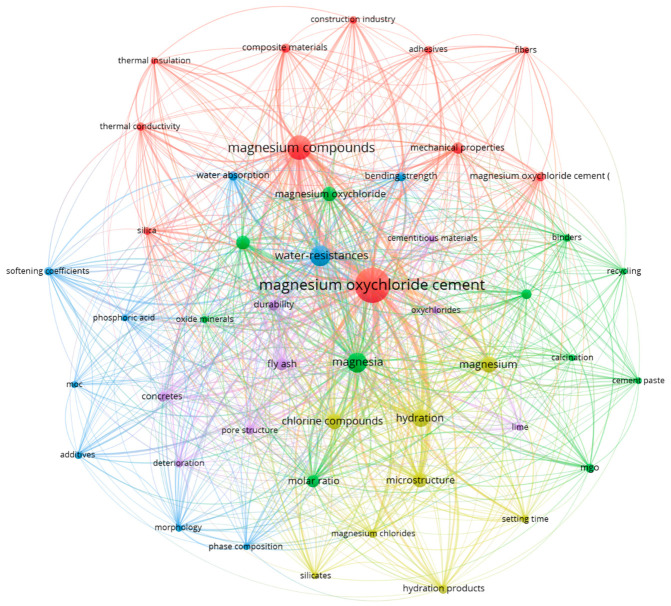
Keyword co-occurrence network of MOC research.

Figure 5 illustrates the keyword co-occurrence network generated using VOSviewer 1.6.20, highlighting the thematic structure of magnesium oxychloride cement (MOC) research. Node size represents keyword frequency, while link thickness indicates the strength of co-occurrence relationships. The central node, “magnesium oxychloride cement,” is strongly connected to major research themes including microstructure, hydration, water resistance, mechanical performance, and magnesium compounds. Distinct clusters reveal dominant research directions: (i) durability and water-resistance enhancement (e.g., softening coefficient, deterioration, and additives); (ii) microstructural and phase characterization (e.g., SEM, XRD, and hydration products); (iii) sustainability and CO_2_-related studies (e.g., carbon dioxide, sustainable development, and recycling); and (iv) composite and performance optimization (e.g., fibers, reinforcement, and bending strength). The dense interconnections among clusters indicate a highly integrated research field linking materials chemistry, durability engineering, and sustainable construction applications.

## 3. Impact of Alternative Cementitious Systems on the Environment

There has been an increase in the production of CO_2_ throughout the last century, with production exceeding the amount of 38 billion tons per annum. Due to such a massive scale, almost 7 to 8% of CO_2_ emissions are attributed to cement production [54]. The research on climate conservation emphasizing technology has placed significant attention on developing plans for implementing technical measures to reduce carbon emissions in cement production. One such roadmap is presented by the International Energy Agency (IEA), which aims to decrease the overall CO_2_ emissions of the cement industry worldwide by 24% by 2050. According to this roadmap, the implementation of alternative materials is responsible for 37% of the reduction in CO_2_ emissions [55]. Although cement substitution is crucial, there is currently a lack of comprehensive evaluations of the environmental impacts of using alternative cement binders. In this context, we consider “alternative cement binders” as binders that can substitute conventional OPC or blended PC binders to some extent but are not in accordance with established PC specifications [56].

**Figure 6:**
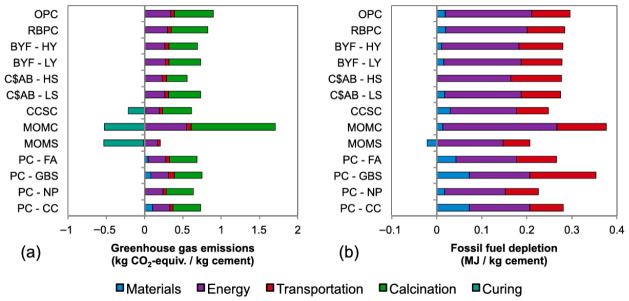
(**a**) Comparative greenhouse gas emission profiles and (**b**) embodied energy demand, expressed in terms of fossil fuel consumption [57].

Miller and Myers [57] conducted a study to measure and contrast the environmental effects that result from manufacturing binders derived from various frequently studied alternative cement systems. These include Belite Yosemite Ferrite Cement, Regular Portland Cement (OPC), Reactive Belite Portland Cement (BYFC), Calcium Sulfoaluminate-Belite Cement (CSBC), Magnesium Oxide Cement (MOC), Carbonate Calcium Silicate Cement (CCSC) in the presence of Mg_2_SiO_4_ (MOMS) and MgCO_3_ (MOMC), and other blended cements. The findings indicate that using the majority of these substitute cements leads to reduced greenhouse gas (GHG) emissions and other environmental impact indicators when compared to PC binders. The amount of reduction depends on factors such as energy consumption during production, emissions generated during clinker formation, and the demand for raw materials. Figure 6 shows the effect of all the cement systems and their energy demands [57].

In order to address the negative environmental impact of cement production, one potential approach incorporates new materials that have been proven to work as a solution, offering both better engineering performance and a lower environmental impact. Examples of such materials include MOC, composite PC-free cement, geopolymer cement, and nanostructured cement, which can be utilized in the construction sector [58].

To boost material effectiveness and work toward the Paris Agreement 1.5 °C target, a study was conducted in Canada to investigate MgO and MOC efficiency and environmental impact panel production. The results indicated that CO_2_ emissions from natural gas-produced MgO were 18% lower than those produced by coal and 11% less than previous studies had reported. Additionally, compared to alternative building envelope walls made of concrete, MOC panels showed a reduced environmental effect, with CO_2_ emissions representing only 78% of ICF emissions and 36% of tilt-up wall emissions, and lower NOx and SOX impacts. MOC panels also exhibited lower human and ecosystem damage impacts compared to concrete-based scenarios and performed similarly to Structural Insulated Panels (SIPs). The addition of 1 kg of MgO to the MOC boards resulted in 1 kWh of energy savings in manufacturing, leading to a 1.4 kg rise in CO_2_ emissions, whereas a 1 kg reduction in overall CO_2_ emissions. Overall, MOC panels were lighter and less harmful to the environment than cement-based alternatives and were comparable to wood-based alternatives, such as SIPs, especially in terms of toxicity. However, their impact was higher than that of conventional stud walls. MOC panels also demonstrated high performance in terms of fire resistance, sound resistance, termite resistance, flood resistance, moisture resistance, and resistance to bacterial damage [59].

## 4. Fundamentals of MOC: Phase Formation and Hydration

### 4.1. Raw Materials

MOC cement is produced using slightly calcined magnesium oxide, along with MgCl_2_ and H_2_O, as its primary components. It is important to remember that the active content of MgO needs to meet specific standards [60,61]. The effectiveness of MOC cement products greatly depends on the quality of MgO. Factors such as particle size, calcination temperature, and MgO activity have a significant impact on how MOC cement performs [62]. It is important to control impurities in raw materials [63]. Alkali metal chlorides quickly absorb moisture, and they do not participate in the hydration process. Instead, they exist freely within the product. The amount of moisture absorbed by the product increases as its chloride concentration rises [64]. When $SO_{4}^{2-}$ ion reacts with MgSO_4_, it creates MgSO_4_, which can significantly weaken the strength and water resistance of the product. Hence, it is crucial to minimize the amount of $SO_{4}^{2-}$ ion present in the product to avoid these adverse effects. This information is found in [63], where the author suggests that the activity level of lightly burned MgO should be moderate and not too high or low. To meet the required standards, MgO must have an activity level of approximately 60% and a fineness of less than 200 mesh. Additionally, the free content of CaO should not exceed 2%, the content of Fe_2_O_3_ should not exceed 1.5%, and the content of $SO_{4}^{2-}$ in MgCl_2_ should be less than 3%.

Excluding certain raw materials can have negative consequences both in the early stages of production, such as product discomfort, heat concentration, warping, and cracking, as well as in the formation of hydrates. The outcome will be a decrease in strength and poor resistance to water, which will ultimately render the products unusable. The proportion of raw materials in MOC is primarily determined by the proportion of active MgO, MgCl_2_, and H_2_O. This ratio is established by considering both actual and theoretical production knowledge formulas [8].

**Figure 7:**
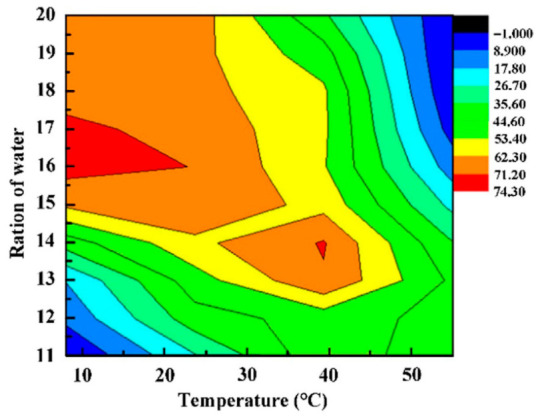
Schematic relationship between temperature and water ratio illustrating the formation domain of phase 5, with color gradients representing its relative percentage content [69].

Advancements in MOC research have led to a more comprehensive understanding of how the performance of raw ingredients has an impact on MOC cement [20,65]. The proportion of MgO/MgCl_2_/H_2_O has a significant impact on MOC cement’s crystal phase composition [66,67]. Mg(OH)_2_ and phase 5 are often created at lower MgCl_2_ concentrations; phase 3 is typically produced in high concentrations of MgCl_2_ solution [68]. It was discovered that phase 3 vanished and phase 5 took its place when the H_2_O/MgCl_2_ molar ratio exceeded 15, showing that the material’s compressive strength as a final product could be altered by adjusting the temperature and MgCl_2_ solution concentration (Figure 7) [69]. Zhou et al. [70] used a thermodynamic model to estimate the types of phases present in MOC cement. They concluded that MOC cement would exhibit superior strength and abrasive resistance if the molar ratio of MgO to MgCl_2_ was within the range of 11 to 17, which would result in a high concentration of phase 5.

There is no definitive rule that specifies the ideal ratio of raw components for MOC cement in practice. The common belief is that the primary objective is to increase the amount of phase 5 content to the maximum level. During the manufacturing process, MgO cannot completely engage while reacting; therefore, MgO/MgCl_2_’s molar proportion needs to be a little above 5. Typically, whenever the ratio of MgO/MgCl_2_ is within the range of 7–9, the resulting products exhibit a superior level of strength. To put it differently, the crystal phase that is formed is dependent on the concentration of MgCl_2_, and a concentration of 28.9% is considered to be a MgCl_2_ solution’s hypothesized concentration at which phase 5 hydrates are present in all forms. Phase 3 is activated if the concentration surpasses this level. Mg(OH)_2_ is the product created when the concentration is less than 28.9%. In contrast, when the MgCl_2_ solution’s concentration increases, the consistency of the concrete slurry becomes thicker, which negatively impacts its workability and may result in incomplete hydration, ultimately leading to reduced strength, halogenation, and other undesirable consequences. In summary, increasing the concentration of MgCl_2_ solution can have detrimental effects on the cement’s performance. To achieve optimal workability and desirable properties for MOC cement, it is recommended to use a slightly lower concentration of MgCl_2_, just below 28.9%. At this concentration, primary hydration aids will be phase 5 with some supplementary Mg(OH)_2_. In summary, the ideal concentration of MgCl_2_ for MOC cement is slightly below 28.9% to obtain the best mixture of workability and overall properties.

### 4.2. Hydration Process

It has become commonly acknowledged in recent times that the process of hydration can be clarified utilizing the kinetics of hydration concepts, and the method changes in terms of mechanism during different stages of hydration [6]. There is a widespread belief that the first stage involves the formation of a gel, which later undergoes crystallization on its surface. In the process of forming specific phases during this period, three stages can be distinguished, namely crystallization, neutralization, and hydroxyl bridging. The equations in [3] can be used to illustrate the three phases involved in the process of hydration, which are:

(1) Stage of neutralization:

(1) $$MgCl_{2}\;\cdot \;6H_{2}O\overset{H_{2}O}{\to }\;{\left[Mg{\left(H_{2}O\right)}_{6}\right]}^{2+}+2Cl^{-}$$

(2) $${\left[Mg{\left(H_{2}O\right)}_{6}\right]}^{2+}\underset{OH^{-}}{\overset{H^{+}}{\to }}\;{\left[Mg\left(OH\right){\left(H_{2}O\right)}_{5}\right]}^{+}+H^{+}$$

(3) $$MgO+H^{+}+5H_{2}O\to \;{\left[Mg\left(OH\right){\left(H_{2}O\right)}_{5}\right]}^{+}$$

During the neutralization process, MgO is mixed with $MgCl_{2}$ solution and reacts with the $H^{+}$ ions present in the solution. This leads to an increase in the concentration of both $Mg^{2+}$ and $OH^{-}$ ions.

(2) Stage of hydroxyl bridging:

(4) $$MxMg^{2+}+\left(z+y\right)H_{2}O\underset{OH^{-}}{\overset{H^{+}}{\to }}\;{\left[Mg_{x}{\left(OH\right)}_{y}{\left(H_{2}O\right)}_{z}\right]}^{2x-y}+yH^{+}$$

The hydroxyl bridging reaction is facilitated by the rising levels of $Mg^{2+}$ and $OH^{-}$. This results in the formation of complex ions of magnesium containing aquohydroxoco groups with multiple nuclei, denoted as ${\left[Mg_{x}\;{\left(OH\right)}_{y}\;{\left(H_{2}O\right)}_{z}\right]}^{2x-y}$.

(3) Stage of crystallization:

(5) $${\left[Mg_{x}{\left(OH\right)}_{y}{\left(H_{2}O\right)}_{z}\right]}^{2x-y}+mOH^{-}+\left(2p-q\right)Cl^{-}+kH_{2}O\to \left(x-p\right)Mg^{2+}+{\left[Mg_{p}{\left(OH\right)}_{q}{\left(H_{2}O\right)}_{r}\right]}^{2p-q}\left(2p-q\right)Cl^{-}\;\cdot \;\left(k+z-r\right)H_{2}O+\left(y+m-q\right)OH^{-}$$

The variables x, y, z, m, k, p, q, and r are the parameters used in Equations (4) and (5). If p = 6, q = 10, and k + z−r = 8, ${\left[Mg_{p}{\left(OH\right)}_{q}{\left(H_{2}O\right)}_{r}\right]}^{2p-q}\left(2p-q\right)Cl^{-}\cdot \left(k+z-r\right)H_{2}O$ can be expressed as ${\left[Mg_{6}{\left(OH\right)}_{10}\right]}^{2+}2Cl^{-}\cdot 8H_{2}O$, which is another expression of $5Mg{\left(OH\right)}_{2}\cdot MgCl_{2}\cdot 8H_{2}O$ (phase 5). ${\left[Mg_{x}{\left(OH\right)}_{y}{\left(H_{2}O\right)}_{z}\right]}^{2x-y}$. The attraction between $Cl^{-}$ and $H_{2}O$ causes them to form gels that are cross-linked and amorphous. These gels eventually transform into crystals. As time goes on and the MOC cement becomes harder, the gaps in its network structure are slowly occupied by crystallized substances, leading to a steady enhancement in its overall performance.

### 4.3. Processing and Curing Effects on Phase Formation and Stability

Cement MOC production is significantly related to hydration, mechanical characteristics, microstructure, and the composition procedure of the crystal phase. These key aspects of MOC cement have a direct connection to the way in which it is manufactured [70,71]. Researchers discovered that by increasing the stirring speed of MOC, they could substantially alter its surface viscosity. They also found that the ideal mechanical properties were achieved when the product was stirred at a speed of 280 rpm for 25 min. If the mixing time is too short or the mixing is uneven, it can result in the production of bubbles, which can cause a degradation in the mechanical properties of the final product. Inadequate mixing can cause the production of bubbles, which can harm the mechanical properties of the product. In addition, if mixing is done excessively, it can lead to a decline in the product’s workability. Another way to say this is that over-mixing can cause the workability of the product to deteriorate. To enhance the mechanical properties of a mixture of MgCl_2_ solution and MgO powder, it is important to control the stirring speed and time effectively. This ensures that the mixture is well-mixed, reducing the free water present and preventing the development of holes, which can make the microstructure better in the mixture. Abdel-Gawwad & Khalil [24] used a new method for manufacturing MOC cement using a one-part process. This method involves controlling the rates of neutralization, hydrolysis, bridging, and condensation during the formation of MOC cement.

The optimal performance of MOC was achieved when the molar ratio of MgO/MgCl_2_ was 14, and the ratio of H_2_O/MgO was 0.1 [72]. The crystal structures are made up of hydrates and are highly susceptible to changes in the surrounding temperature and humidity. The creation of crystal phases and microstructure in cement products is significantly impacted by the temperature and humidity conditions during the curing process [73]. Typically, the item needs to be cured in an environment that is either free of moisture or kept separate from the surrounding air [74]. At lower temperatures ranging from (8–25) degrees Celsius, when the ratio of H_2_O/MgCl_2_ is approximately 13, phase 5 is present in significant amounts while phase 3 is almost absent [69]. Phase 3 will emerge with a minor increase in the concentration of MgCl_2_ solution; phase 3 is more steadfast than phase 5, despite both phases being metastable crystals [68]. Nonetheless, whenever the temperature surpasses a certain threshold, the production of by-products such as brucite will occur, leading to a decline in performance. The formation of brucite requires a high activation energy, and the temperature plays a significant role in its formation. In general, brucite is more likely to form at greater temperatures because a high temperature helps the high activation energy reaction. Additionally, phase 5 activation energy is larger than that needed for the production of brucite [75]. Phase 5 will release 2 mol of water when the temperature goes beyond 72 degrees Celsius [11]. When the temperature exceeds 80 degrees Celsius, the integrity of phase 3’s structure will be compromised [76]. MOC cement can achieve high temperatures without external heating due to the substantial heat output from the hydration process. This has piqued the interest of researchers who want to study the crystallization of MOC cement at high temperatures. The authors of [77] identified the 2-1-2 phase and 3-1-1 phase after examining the elements of MOC cement between the temperatures of 50 and 150 degrees Celsius. At a temperature of 120 °C, Ref. [78] identified the formation of three phases: 9-1-4, 2-1-4, and 2-1-2. However, these phases were determined to be unstable at room temperature. The literature [79] confirmed that during hydration, the 9-1-4 phase undergoes a quick transformation into phase 3, which is comparatively more stable. Table 1 displays a succinct overview of the hydrated stages under varying temperatures.

MOC cement’s phase 5 is widely regarded as the most superior crystal phase due to its exceptional mechanical characteristics. In order to create high-quality MOC cement, it is necessary to maximize the content of phase 5. As per the literature [74], it is stated that when the temperature for the curing process is maintained at a low temperature of 25 °C during the initial stage and then gradually increased to a higher temperature of 70 °C during the middle and later stages, it helps in promoting the growth of phase 5. Consequently, the resulting MOC cement product will exhibit exceptional mechanical properties.

**Figure 8:**
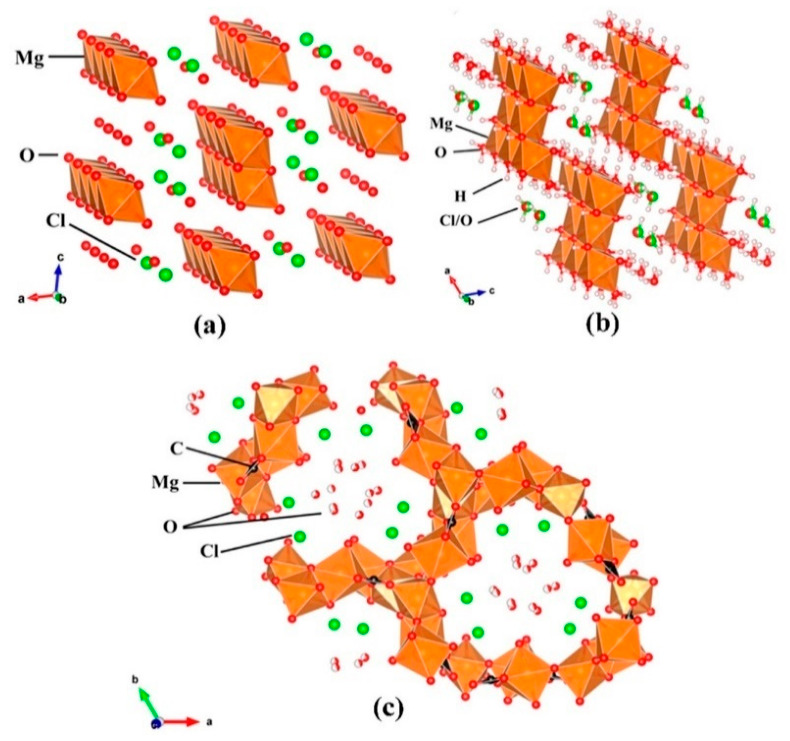
Crystal structures of (**a**) phase 3, (**b**) phase 5, and (**c**) chlorocarbonate phases. Hydrogen atom positions are not shown in panels (**a**,**c**) [11].

## 5. Properties of MOC

The hydration process, mechanical characteristics, and crystal shape of MOC cement underwent changes through different alterations, resulting in enhancements in the material’s properties. In order to investigate the process of modification, the scientists thoroughly analyzed the micro and macro features of MOC cement, with the goal of producing materials that exhibit superior performance.

### 5.1. Fresh-State Properties

MOC often exhibits rapid stiffening and pronounced exothermicity because hydration involves fast dissolution of MgO in MgCl_2_ brine, followed by precipitation and intergrowth of magnesium hydroxychloride phases. Fresh-state properties therefore directly reflect early hydration kinetics and evolving particle–hydrate networks, rather than being purely “handling” artifacts. Recent studies consistently point to three coupled controls of workability, setting, and heat release: (i) mix chemistry and MgO reactivity/impurities; (ii) mixing-driven dispersion and mass transfer; and (iii) kinetic regulation by admixtures and mineral additions.

**Figure 9:**
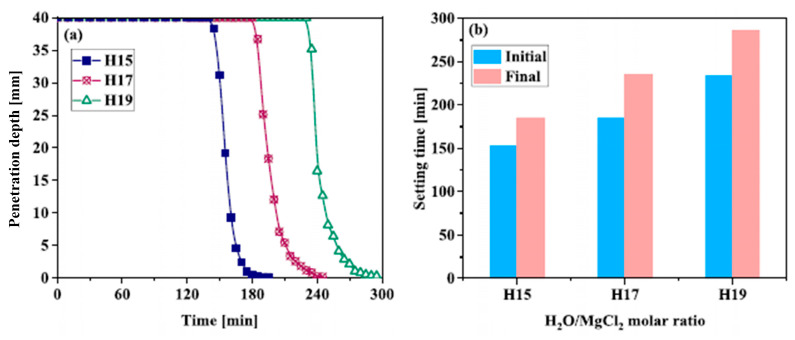
(**a**) Evolution of the penetration of the Vicat needle with time; (**b**) setting time of MOC cement with a varying H_2_O/MgCl_2_ molar ratio [81].

He et al. [3] showed that both initial and final setting times decreased with increasing mixing time and stirring rate, with an approximately linear relationship between mixing duration and setting time. The practical implication is a trade-off, as higher shear improves homogeneity but can accelerate neutralization and ion build-up, thereby shortening setting time. Because MOC is sensitive to such processing variables, cross-study comparisons require explicit reporting of mixing protocol (speed, duration, and sequencing). Rheology in MOC evolves rapidly and nonlinearly as hydration products nucleate and percolate. Yuan et al. [81] quantified time-dependent rheology under different H_2_O/MgCl_2_ ratios and proposed a staged setting mechanism in which early colloidal interactions and subsequent hydrate precipitation progressively increase yield stress and viscosity. The penetration depth data captured inflection points during stiffening as shown in Figure 9, while isothermal calorimetry showed concurrent changes in heat flow behavior supporting a direct coupling between rheological build-up and exothermic hydration. Notably, the water ratio is not merely a “workability parameter”; it shifts both the timing and intensity of structure build-up and heat release, so ratio and temperature must be harmonized when interpreting setting or workability trends.

**Figure 10:**
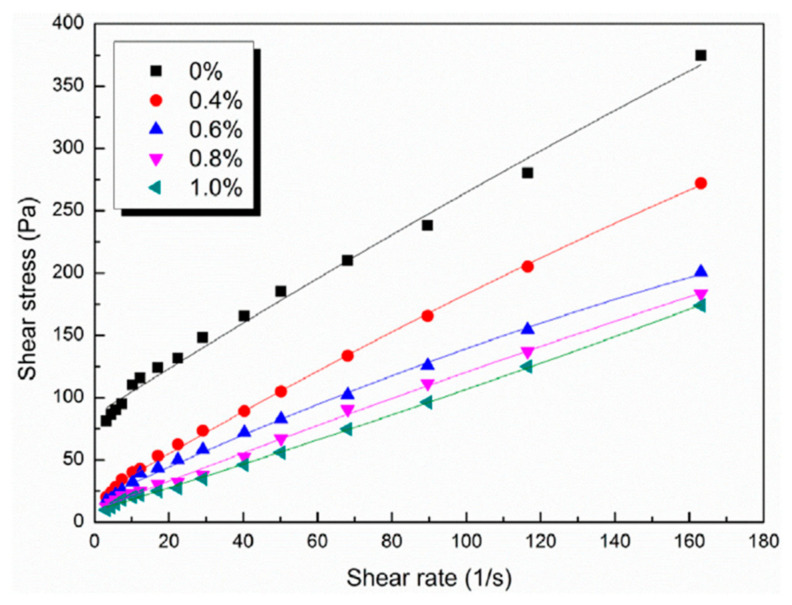
Rheological variations with increasing PCE content [82].

Admixture strategies that stabilize workability generally act by delaying flocculation and/or precipitation. Polycarboxylate superplasticizer (PCE) reduced yield stress and plastic viscosity in MOC paste via enhanced dispersion, as shown in Figure 10 [82]. However, Wu et al. [82] also showed that PCE modifies early hydration behavior (tracked by electrodeless resistivity and microstructural evidence), which is consistent with the delayed formation of binding products that otherwise drive rapid stiffening. Thus, improved flow is inseparable from kinetic retardation, and dosage windows should be evaluated alongside mixture ratios and target setting times.

Organic acids offer another route to workability retention by complexing Mg^2+^ and moderating precipitation. Guan et al. [83] reported that citric acid increased setting times and reduced the rate at which yield stress and plastic viscosity rose over time, demonstrating that “initial fluidity” alone is insufficient for MOC; time-resolved rheology is needed to capture rapid structural build-up. In foamed MOC composites, sodium citrate was used to regulate hydration and microstructure development by delaying hydration through chelation effects [84], illustrating the relevance of citrate-type modifiers when premature stiffening would destabilize foam or induce placing defects. Mineral additions can affect flow, setting, and thermal evolution through dilution, micro-filling, and altered nucleation. Using isothermal calorimetry, Feng et al. [85] showed that industrial waste incorporation changed heat flow profiles and cumulative heat evolution of MOC, indicating modified kinetics illustrated in [85]. A clearer combined workability–setting response was reported for fly ash; Xie et al. [86] found that fly ash increased fluidity (1.3–11.4%) and extended initial setting time (up to 26.4%) relative to a control mix, with calorimetry capturing associated changes in heat evolution. These results position fly ash as a dual-function modifier (handling plus kinetic moderation), but also show that benefits are incremental and mixture-dependent.

Fresh-state constraints become especially stringent in fiber-reinforced and strain-hardening MOC composites, where adequate flow must coexist with fiber dispersion. Hybrid fiber reinforcement reduced flowability and changed rheological parameters, indicating that fiber type and dosage are first-order drivers of workability loss [87]. Yu et al. [88] reported that a tailored MOC-based strain-hardening composite achieved a flow-table spread of 240 mm without a high-range water reducer and maintained initial/final setting times of 5.2 h/7.8 h, showing that powder packing and water balance can partially offset rheological penalties in high-performance mixes.

**Figure 11:**
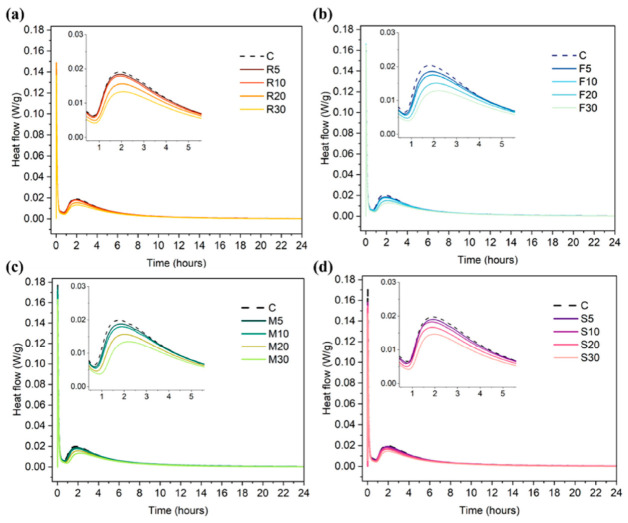
Variations in the heat flow with incorporation of different industrial wastes: (**a**) red mud; (**b**) fly ash; (**c**) metakaolin; and (**d**) slag.

Finally, early hydrate assemblage depends on raw material chemistry. Cui et al. [89] demonstrated that free CaO content significantly affects the phase composition of MOC, implying that impurity control and reporting are essential for interpreting differences in setting and heat evolution. Because studies employ different workability metrics (e.g., spread/flow tests versus rheometer protocols) and setting definitions, direct numerical comparison remains challenging; nevertheless, the convergence of qualitative trends strengthens mechanistic interpretation. Overall, fresh-state optimization in MOC is best treated as a coupled dispersion–kinetics problem: improving workability commonly requires slowing the same reactions that deliver rapid setting and early strength. For comparability and translation, studies should report MgO reactivity/impurities, MgO/MgCl_2_/H_2_O ratios, mixing protocol, temperature, time-resolved fluidity or rheology, Vicat (or equivalent) setting times, and calorimetry-based heat flow curves (Figure 11).

### 5.2. Microstructure and Hydration Phases

In addition to the material’s phase composition, the microstructure of MOC cement plays a vital role in determining its macroscopic properties [90]. The properties at the macroscopic level, such as porosity and strength, are a reflection of the microscopic attributes of materials. Studying the microstructure of MOC cement composites is advantageous in order to understand the underlying mechanisms.

#### 5.2.1. SEM

MOC is an inorganic compound with the chemical formula Mg(OH)Cl. MOC is a white, crystalline, water-insoluble powder used as a cement binder and in producing various construction materials. In the research on MOC cement, to examine the morphological traits of the crystal phase, scanning electron microscopy (SEM) is most commonly used. Tooper and Cartz [91] utilized SEM images to observe phase 5’s rod-like shape, claiming that the acicular crystal’s cross-section was strengthened. Matkovic et al. [92] confirmed that when viewed via SEM, the crystal phase of MOC cement typically exhibited an acicular shape. Typically, the crystal phases join with one another and compactly form flake crystals, whereas the only phases that exhibit a needle shape are those that are in the voids. Chau and Li [19] studied MOC cement crystals with different combinations of raw materials and discovered that acicular crystals had tiny particles on their surface when viewed under a microscope. They discovered that when the MgO content was low, the crystal phase had an irregular shape. On the other hand, at a molar ratio of MgO/MgCl_2_/H_2_O, the crystal shape and mechanical characteristics were at their optimum; at a microscopic level, the crystal phase was both uniform and dense. This was achieved with a 6.8/1/10 ratio. An inorganic modifier that shows potential in increasing water resistance is phosphoric acid. Researchers have found that crystals with a needle-like appearance were visible on the surface of MOC cement when it was treated with phosphoric acid, whereas samples without phosphoric acid had an amorphous or gel-like surface morphology [23]. As a complementary analysis to SEM, the EDX test is commonly utilized to quantify the element content of crystals [19]. However, it is observed that hydrogen atoms may not be picked up by EDX since the EDX instrument is insensitive to hydrogen atoms.

**Figure 12:**
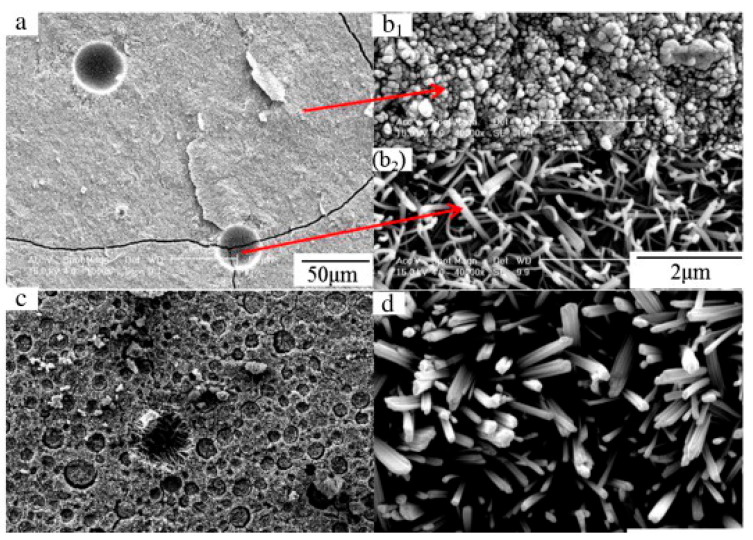
The images show the surface structure of MOC cement: (**a**,**b_1_**,**b_2_**) represent the cement without $H_{3}PO_{4}$, while (**c**,**d**) represent the cement with 0.5 M $H_{3}PO_{4}$ [23].

Evidently, the presence of phosphoric acid on the surface of MOC cement results in the formation of needle-shaped crystals, whereas without it, the surface appears amorphous or gel-like, as shown in Figure 12 [23].

In addition, the EDX test is commonly utilized as a complementary analysis to SEM to measure the elemental composition of crystals in a quantitative manner [19]. Hu et al. [93] employed EDX to examine the elemental composition of phase 5, and the findings suggest that the measured values correspond closely with the theoretical values. It is important to disregard any gold element detected by the analysis, as it may originate from the coating material. The atomic percentage is computed solely based on the signals emitted by magnesium, oxygen, and chlorine elements, as hydrogen atoms cannot be detected by EDX due to the device’s lack of sensitivity to them.

#### 5.2.2. XRD

**Figure 13:**
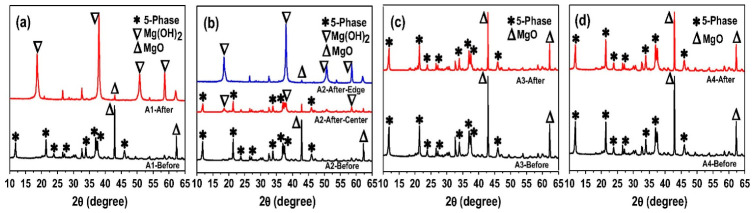
XRD patterns of MOC specimens with varying mass fractions of phase 5 and H_3_PO_4_ before and after 28 days of water immersion: (**a**) (0% phase 5, 0% H_3_PO_4_); (**b**) (1% phase 5, 0% H_3_PO_4_); (**c**) (0% phase 5, 1% H_3_PO_4_); and (**d**) (1% phase 5, 1% H_3_PO_4_) [15].

XRD analysis is a technique that distinguishes between various crystals based on their unique X-ray characteristics, enabling the identification of the phase composition of MOC cement. As presented and illustrated in Figure 13 [15], at a molar ratio of $MgO/MgCl_{2}/H_{2}O$ = 6.5/1/13, the primary constituents of MOC cement were phase 5 and a small quantity of unreacted MgO, with no indication of phase 3. The addition of phosphoric acid to MOC cement yields a notable improvement in water resistance, as evidenced by the nearly unaltered phase composition observed after immersion (Figure 13c,d). In contrast, the absence of phosphoric acid in the sample leads to extensive hydrolysis of phase 5 to Mg ${\left(OH\right)}_{2}$, as demonstrated in Figure 13a,b. Due to the presence of available $H^{+}$ during the initial hydration period, the distinctive peak of the 2-1-2 phase was identified at 30 min of hydration. As time progressed, the 2-1-2 phase changed into phase 5, which is consistent with the previous literature [23,75]. XRD analysis can be used not only to determine the MOC cement phase composition but also to roughly measure the size of phases [19] and quantitatively analyze their content at various stages [94]. This further helps in exploring the crystal characteristics of MOC cement.

#### 5.2.3. Fourier Transform Infrared Spectroscopy (FTIR)

FTIR analysis is a method that can identify crystal phases’ chemical composition in MOC cement and can serve as a criterion for determining whether the modification process was successful. Tan et al. [77] observed that the $H_{3}PO_{4}$ vibration peak was absent in the MOC cement treated with phosphoric acid using FTIR, but they detected the presence of P-OH and $PO_{4}^{3-}$, which suggests that phosphoric acid was fully incorporated into the cement matrix. Phase 5 OH stretching vibrations and Mg(OH)_2_ generate the absorption bands at 3610.3 cm^−1^ and 3698.2 cm^−1^. Phosphoric acid and tartaric acid were then added, and the ratio of the peak at 3610.3 cm^−1^ to 3698.2 cm^−1^ increased significantly, indicating a higher content of phase 5 in TA-MOC and PA-MOC than in N-MOC. Additionally, the oxhydryl bond stretching vibrations produced the absorption bands at 3000–3600 cm^−1^, and the *H*-*O*-*H* bending was attributed to the bands between 1600 and 1640 cm^−1^. To comprehensively evaluate the performance of MOC cement, it is necessary to analyze it from multiple perspectives, including the macro and micro levels. This helps to provide sufficient evidence for the enhancement of material performance. In order to achieve a more comprehensive characterization, researchers are continuously coming up with innovative research methods that are efficient and reliable. For instance, Dorrepaal and Gowen [95] developed a method that utilizes a combination of Raman chemical mapping and near-infrared (NIR) hyperspectral chemical imaging to investigate the heterogeneity of MOC cement.

### 5.3. Mechanical Properties (Flexural Strength and Remaining Properties)

**Figure 14:**
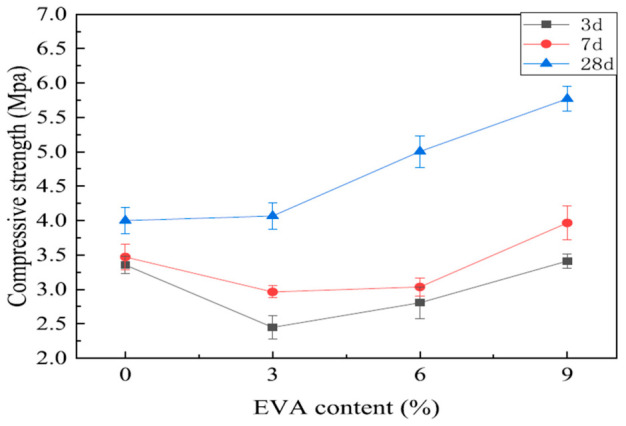
Compressive strength with varying percentages of EVA [97].

Magnesium oxychloride cement (MOC) is typically characterized by high early and ultimate compressive strength, but its mechanical response is highly sensitive to hydrate assemblage (phase 5-dominated systems), pore structure and fillers, and exposure/curing history. The literature highlights a consistent pattern where strength is maximized when phase 5 interlocking networks are preserved and pores are refined, while toughness and flexural performance are best improved through hybrid mechanisms (polymer film formation, fiber bridging, or Mg–Si gel development) [96,97,98,99,100]. The ratio of raw materials, curing conditions, and the incorporation of modifiers have a considerable impact on the compressive strength of MOC cement. For a paste-like MOC at ambient condition, Chang et al. [96] reported 113.3 MPa compressive strength with a phase 5-rich needle-like microstructure, confirming the dominant contribution of phase 5 to compressive load transfer. Similarly, rice husk ash (RHA)-modified MOC mixtures achieved a broad high-strength range ( ≈ 90–120 MPa), with an optimum at 20% RHA giving 121.5 MPa (28 d); higher replacements reduced strength to 81 MPa at 50% RHA, indicating a classic dilution–densification trade-off [100]. Similarly, by adjusting the curing conditions in a reasonable way, the MOC cement’s compressive strength can be increased up to approximately 150 MPa. Adding fibers to MOC cement can improve its toughness, resulting in an increase in flexural strength, while the effect on compressive strength is not significant [101,102]. It is important to emphasize that when additives are employed for modifying MOC cement, a moderate amount of additives can be helpful in enhancing the strength, but an excessive amount of additives can lead to a significant reduction in the strength. Additionally, it should be noted that the loading mode of a test can significantly affect the results of strength measurements due to the obvious rate response characteristic of stress. Therefore, it is important for studies to investigate strength under the same loading mode to ensure that results can be directly compared. Previous research has explored strength under various loading rates, but direct comparisons cannot be made without consistent loading modes [23,69,94]. For foam–concrete systems, compressive strength is governed by pore architecture and stabilization of the binding hydrates. In MOC foam concrete, EVA polymer addition was reported to increase 28-day compressive strength by 44.2% at 9% EVA (vs. 0% EVA), as shown in Figure 14. This demonstrated that polymer film continuity can offset porosity-driven losses by strengthening the solid skeleton and controlling hydrate decomposition [97]. Additionally, MOC cement is known for its rapid hardening property, and its strength growth rate decreases as time goes by. The strength attained within the first 7 days is around 90% of the strength achieved in 28 days [74]. After 28 days, the strength remains relatively constant.

Flexural capacity is particularly sensitive to microcrack arrest and interfacial bonding. In “pure” MOC, a flexural strength of 18.4 MPa is reported at room temperature [96]. However, thermal exposure causes disproportionate flexural loss: at 100 °C, flexural strength decreases by 57.07% primarily due to the volatilization of gel water that connects gel/phase5 networks, while compressive strength changes far less at that stage [96]. This highlights that flexure is more sensitive than compression to microstructural “connectors” (gel water, inter-crystal bridges), whereas compression can still be partially sustained by remaining solid phases even after partial phase 5 degradation. In fiber cement products, Gomes et al. [98] showed that rice husk silica (RHS) improves flexural behavior, increasing modulus of rupture (MOR) and toughness via filler effects and Mg–Si hydrate (M-S-H)-type gel formation, with performance evaluated in dry, saturated, and warm-water aged states. Reported baseline MOR values for unmodified systems reached 6.38 MPa (dried condition), and RHS generally improved saturated behavior compared with unmodified matrices, supporting the role of micro-filler packing plus secondary gel formation in bending resistance [98].

### 5.4. Durability Aspects

Durability remains the principal constraint on the broader deployment of MOC in construction because the strength-giving oxychloride hydrates (notably “phase 5”) progressively destabilize when water penetrates the pore network, promoting hydrolysis, brucite formation, loss of cohesion, and in some cases, accelerated carbonation under humid exposure [99,103,104]. For this reason, durability design for MOC should be treated as a coupled problem of residual strength after wet exposure and pore structure/transport pathways that govern water ingress and reaction kinetics.

A practical macro-indicator of water durability is the water-resistance (softening/strength retention) coefficient, commonly expressed as the ratio of compressive strength after soaking to that before soaking as given in Equation (6). This concept aligns directly with “strength retention coefficient” approaches used in recent MOC and MOC-paste durability studies [103,105]. A higher softening coefficient is indicated by a lower loss of strength after soaking in water. The softening coefficient may be expressed using formula [21,22]:

(6) $$R_{f}=R_{x}/R_{o}$$

where the softening coefficient is $R_{f}$; after soaking, $R_{x}$ is the compressive strength; and Ro stands for compressive strength prior to soaking. By using water-resistant modification methods, the MOC cement capacity for resistance to water may be significantly improved. Currently, the most effective way to achieve this is through the use of a compound modifier that includes polymer and phosphate materials [106]. The literature highlights why unmodified or “pore-coarsened” MOC loses strength rapidly. In MOC incorporating form-stable phase change material (FSPCM), the pore system is dominated by micropores (0.01–0.1 μm) comprising ~80% of voids, creating continuous pathways for water molecules; the microstructure becomes less compact, and mechanical properties decline accordingly [107]. Similarly, when porosity is intentionally increased (e.g., by cenospheres), thermal insulation can improve, but the durability penalty is an expanded connected pore network unless compensating densification occurs [39]. Even for chemical modifiers, pore redistribution matters: tartaric acid and phosphoric acid slightly increased porosity and produced a pore system dominated by small capillary pores (10–100 nm; 71.9%)**;** this range still supports water transport, implying that any water-resistance gains must be attributed to phase/chemistry effects and not simply reduced total porosity [108].

By contrast, the strongest durability gains appear when pore refinement and gel formation occur together. A clear benchmark is the silica fume–fly ash hybrid approach, where the most water-resistant formulation reported retained compressive strength at 100% after 28 days and 95% after 56 days of immersion [103]. Importantly, this exceptional retention coincided with an optimized pore volume distribution: the SF15–FA15% system exhibited a comparatively high fraction of gel-scale pores and the lowest capillary pore fraction (50–5000 nm) of 4.16%, indicating a microstructure that restricts transport while maintaining binding continuity [103]. This provides a mechanistic durability pathway: durability improves most reliably when pore connectivity is shifted away from capillary-dominated transport toward gel-dominated, discontinuous pathways, while simultaneously forming stabilizing amorphous/gel products [103].

Phosphate-based modification remains one of the most consistently validated routes to improved wet durability, but its performance is sensitive to mixing/curing practices and does not uniformly resolve all durability modes. Sodium monofluorophosphate (MFP), phosphoric acid (H_3_PO_4_), and KH_2_PO_4_ all markedly increased compressive strength retention in water; the highest values reported include 0.98 (28 d) and 0.95 (56 d) for 1% H_3_PO_4_ and 0.95 for 1% MFP [99]. However, flexural strength retention improved far less, suggesting that phosphate modification preferentially protects compressive load-bearing integrity rather than fully suppressing crack-/interface-controlled degradation modes. Volume stability is also a durability dimension: phosphate/MFP additions substantially reduced immersion-induced volume change compared with the control, and curing under a plastic film cover was recommended to limit microstructural disruption [99].

A complementary and highly practical strategy is high-volume gypsum modification in magnesium oxychloride cement paste (MOCP). When 80% flue gas desulfurization gypsum (FG) or 80% phosphogypsum (PG) (by weight of MgO) was incorporated, the 28-day water-immersion strength retention coefficient (SRC) increased to 61.02% (FG) and 46.55% (PG), compared with 28.99% for the control and only 8.41% for 80% natural gypsum [105]. This comparative outcome is important: natural gypsum coarsened pores and accelerated water erosion, whereas waste gypsum improved phase 5 stability in water and reduced deleterious residual soluble chloride salt, providing a more durable hydration environment. The same study also shows that durability is intertwined with dimensional stability, as reducing brucite-forming potential improved volume stability for high-volume gypsum mixes [105]. Collectively, the phosphate and waste gypsum evidence supports a durability principle: effective wet durability requires controlling both phase stability (reducing hydrolysable pathways) and transport/chemistry (pH/ion environment and pore connectivity) [99,105].

Long-term field evidence shows why “short-term water resistance” does not fully equal service-life durability. Phosphoric acid-modified MOC sampled after 2–16 years of natural exposure exhibited environment-dependent microstructures: humid exposure produced loose, porous morphologies, whereas dry air produced dense matrices but with drying shrinkage cracks [104]. Carbonation phases forming near surfaces were reported as potentially beneficial to internal stability, while overall phase evolution trends toward carbonate-bearing assemblages over time, with water availability being a prerequisite for both hydrolysis and carbonation progression. Therefore, durability design must explicitly distinguish immersion/wet cycling from humid air exposure and must consider the possibility that “protective” surface carbonation may coexist with internal decomposition where moisture persists [104].

For structural applications, reinforcement durability is an additional limiting factor. Organic-coated reinforced MOC exposed to aggressive salt-lake-type conditions showed slow electrochemical deterioration: the corrosion potential decreased from −0.55 to −0.68 V over 2610 days, while the corrosion current density remained within a “no corrosion” regime (0.0342–0.071 μA/cm^2^) for coated steel, demonstrating the shielding value of organic coatings [109]. A multi-factor degradation model projected a damage state at approximately 12,450 days, underscoring that reinforcement compatibility can be managed, but must be treated as a separate durability design layer beyond matrix water resistance alone [109].

### 5.5. Functional Properties

Functional properties, including thermal insulation, fire performance, and surface wear resistance are often cited as key motivations for using MOC in boards and specialty composites. Recent studies indicate that these benefits are tightly controlled by pore architecture and the moisture sensitivity of chloride-bearing hydrates; therefore, functional performance must be discussed alongside environmental conditioning and microstructural stability [110,111].

Lowering thermal conductivity is achieved mainly by deliberately increasing stable porosity, either through chemical foaming or through ultra-lightweight composite design. Hao and Li [97] quantified this trade-off in modified MOC foam concrete. Raising the hydrogen peroxide (H_2_O_2_) dosage from 1% to 4% reduced the thermal conductivity from ~0.239 to ~0.059 W/(m·K) while decreasing the oven-dry density from ~832 to ~353 kg/m^3^. The same change reduced the compressive strength and lowered the softening coefficient after soaking [97]. Their orthogonal analysis further identified H_2_O_2_ content as the dominant factor governing thermal conductivity, whereas the MgO/MgCl_2_ ratio, fly ash, and EVA powder had smaller single-factor effects. Functionally, this implies that in foam-type MOC, insulation performance is primarily a pore volume problem; additives become most valuable when they stabilize the pore wall, limit coalescence, and mitigate the durability penalty associated with high porosity. Similarly, Davraz, Koru, and Akdağ [112] prepared an ultra-lightweight MOC composite (L·MOC) targeting density ≤ 150 kg/m^3^ and conductivity ≤ 0.05 W/(m·K). An optimized mixture achieved a density of ~101 kg/m^3^, a compressive strength of ~192 kPa, and a thermal conductivity of 0.0478 W/(m·K). Compared with Hao and Li [97], this strategy pushes conductivity lower by further reducing density, but the strength level shifts intended use toward insulation cores and non-loadbearing panels. Together, these studies define a practical design parameter at the expense of mechanical performance and water resistance.

Moisture effects are a key confounder. Aiken et al. [110] showed that magnesium oxychloride boards can exhibit high-humidity dimensional instability and “crying” (liquid exudation), which would be expected to increase apparent conductivity and accelerate degradation in service. Similarly, applications in saline environments emphasize the importance of conditioning protocols when functional properties are claimed [113,114]. Although Guo et al. [111] demonstrated that fly ash can improve mechanical performance under water attack, comparable evidence linking conditioning state (dry/wet cycles, RH exposure) to thermal conductivity remains limited. MOC is widely perceived as fire resistant because it is inorganic and contains bound water, but its performance is controlled by hydrate stability. Jiříčková et al. [115] examined the thermal stability of phase 5 (5Mg(OH)_2_·MgCl_2_·8H_2_O) and concluded that disruptive decomposition processes are completed below ~470 °C, accompanied by the release of water and hydrochloric acid. This mechanistic picture supports moderate-temperature endothermic dehydration but also indicates likely microstructural destabilization as temperature rises.

At the composite scale, Rawat et al. [116] measured residual properties of hybrid fiber-reinforced MOC after heating. At 200 °C, mixes containing polypropylene (PP) fibers gained compressive strength (normalized residual strength > 1), while a mix without PP decreased (residual ratio ~0.73), which is consistent with drying, continued reactions, and PP-enabled pressure relief pathways. At 400 °C, in a fiber-controlled retention system, a basalt-only mix retained ~30% strength, whereas a basalt + PP hybrid mix retained ~88%. At 600 °C, mass loss approached ~31%, and residual compressive strengths fell to ~9–14 MPa; at 800 °C, strengths were negligible (~1–2 MPa) [116]. Therefore, “fire resistance” should be reported as residual strength–mass loss–damage profiles versus specified temperature–time histories, rather than as a qualitative advantage.

## 6. Property Enhancement of MOC by Using Additives

Employing appropriate raw materials and utilizing reasonable production and curing methods can effectively ensure that MOC cement products possess satisfactory mechanical characteristics. Although the previously mentioned methods can address various issues with MOC cement, the significant flaw of low water resistance cannot be eradicated. Equations (7) and (8)describe the process of decomposition that occurs in a high moisture environment for phases 5 and 3 [117].

(7) $$5Mg{\left(OH\right)}_{2}\cdot MgCl_{2}\cdot 8H_{2}O\to 5Mg{\left(OH\right)}_{2}+Mg^{2+}+2Cl^{-}+8H_{2}O$$

(8) $$3Mg{\left(OH\right)}_{2}\cdot MgCl_{2}\cdot 8H_{2}O\to 3Mg{\left(OH\right)}_{2}+Mg^{2+}+2Cl^{-}+8H_{2}O$$

The equations presented above demonstrate that the breaking down of phases 5 and 3 leads to the creation of brucite that has a less tightly organized structure. MOC cement performance has significantly declined due to the poor performance of brucite, which is much worse than phases 5 and 3. It was noted that the salts corresponding to phases 3 and 5 possess the characteristics of being a strong acid and a weak base [118]. When in contact with water, these substances will break down and create a small quantity of hydrochloric acid. This acid will then dissolve some of the brucite, resulting in the formation of soluble magnesium chloride. As a consequence, the structure and composition of MOC cement will be harmed, and its overall effectiveness will be significantly reduced. In addition, if the MOC cement is placed in hot water, its effectiveness declines at a faster and more severe rate [119].

Enhancing the ability of MOC cement to resist water is still a persistent difficulty in this area, and the prevalent approach involves incorporating additives to modify the compound [120]. The substances that primarily enhance water resistance are divided into two categories: inorganic additives and organic additives. Reinforcing fiber is an excellent modifier to use if there is a need to improve the mechanical properties. In order to select the appropriate additives based on performance criteria, it is important to consider the impact of factors like the quantity of doping, particle size, and pH on the final product.

### 6.1. Inorganic Additives

Inorganic admixtures of phosphate and minerals, such as phosphoric acid, are commonly mixed with MOC cement as microadditives to enhance its water resistance. These additives are effective in improving water resistance. The authors of [117] conducted a study on the impact of adding a little amount of phosphate that is soluble to MOC cement. Deng found that the addition of 0.74 wt% H_3_PO_4_ resulted in a strength retention rate of 96%, while the rate without H_3_PO_4_ was only 6.4%. The study demonstrated that adding soluble phosphate to MOC cement can significantly improve its strength retention rate. Chen’s [121] research on the impact on the crystal phase of phosphoric acid, which indicated that the addition of 1 wt% of phosphoric acid by weight transformed into a thin, rounded crystal from a dense columnar crystal shape, and phase 5’s corners and edges became blurred. Phosphoric acid can change the crystal phase and its morphology. Li et al. [122] highlighted that the addition of KH_2_PO_4_ to MOC cement resulted in the formation of a gel-like phase known as phase 5. The study also found that the level of phase 5 generated was directly proportional to the cement’s resistance to water, meaning that higher levels of phase 5 resulted in better water resistance. Zhang et al.’s [15] research involved the addition of phosphoric acid and a 5-phase seed crystal simultaneously to prepare MOC cement. This approach led to an improvement in both the strength and water resistance of the cement, achieving the goal of enhancing both properties at the same time.

Inclusion of phosphate ions is the main reason why phosphoric acid is effective in enhancing the resistance of MOC cement to water. In other words, due to the presence of phosphate ions, adding phosphoric acid to MOC cement can result in an increase in water resistance. According to the claim, the formation of insoluble phosphate can provide protection to phase 5 by preventing it from being hydrolyzed. In other words, the assertion is that the formation of insoluble phosphate serves as a protective barrier for phase 5 against hydrolysis [123]. If you mix magnesium chloride with phosphoric acid, the phosphoric acid will undergo ionization, as stated in Reference [106]:

(9) $$5H_{3}PO_{4}⇄H_{2}PO_{4}^{-}+H^{+}$$

(10) $$H_{2}PO_{4}^{-}⇄HPO_{4}^{2-}+H^{+}$$

(11) $$HPO_{4}^{2-}⇄PO_{4}^{3-}+H^{+}$$

When there is $Mg^{2+}$ in a solution, it has a tendency to react with $HPO_{4}^{2-}$, resulting in the creation of a highly water-resistant and stable phase known as $MgPO_{4}\cdot 3H_{2}O$ [83]. While $MgPO_{4}\cdot 3H_{2}O$ is present, it can modify the points of contact between the phases to strengthen the adhesive force, resulting in increased compactness and improved resistance of MOC cement to water [23]. The way in which soluble phosphate enhances the water resistance of MOC cement is comparable to how phosphoric acid functions. However, according to Dinnebier et al. [78], insoluble phosphate was not detected in the XRD test. He suggested that the amount of $Mg^{2+}$ in the solution may decrease due to phosphate ions, which in turn makes the primary water phases, phases 3 and 5, of the crystals more stable. Therefore, it is necessary to conduct further research on the altered mechanism of phosphate. Apart from inorganic acid, certain organic acids like citric acid [124] and tartaric acid [108,125] have demonstrated their ability to enhance water resistance.

Mineral admixtures typically denote materials that include fly ash and silica glass, which are examples of active $SiO_{2}$ powder, among others [126,127]. The term active $SiO_{2}$ pertains to $SiO_{2}$ that has a significant surface area, and can combine with MgO to produce silicates that are insoluble, namely ($3MgO\cdot 4SiO_{2}\cdot H_{2}O\;\mathrm{and}\;MgSiO_{3}$) and MgSiO_3_ [83]. Equations 12 and 13 depict the reactions that are pertinent to the topic. The active proportion refers to the amount of $SiO_{2}$ that is capable of taking part in a reaction. The excess $Mg^{2+}$ can be consumed by the reaction, while insoluble silicates can plug the pores between crystals and decrease the amount of soluble $Mg^{2+}$. As a result, this can lead to a reduction in porosity and an improvement in the compactness of the cement [128,129].

(12) $$3MgO+4SiO_{2}\left(active\right)+H_{2}O\to 3MgO\cdot 4SiO_{2}\cdot H_{2}O$$

(13) $$Mg^{2+}SiO_{2}\left(active\right)+2OH^{-}\to MgSiO_{3}+H_{2}O$$

**Figure 15:**
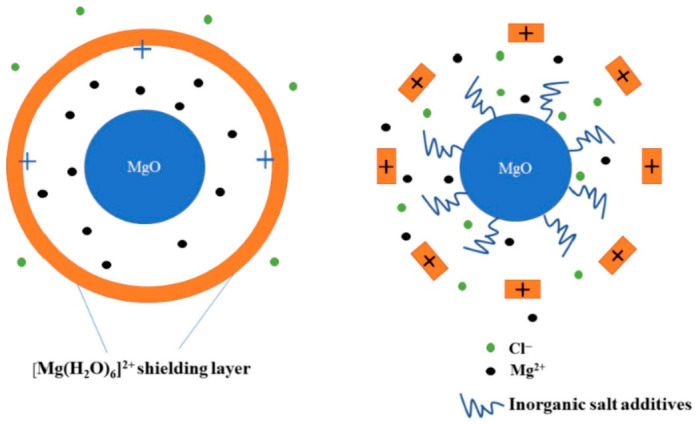
Hydration process of MOC cement with inorganic salt additives [130].

Figure 15 illustrates the role of inorganic salts in promoting low-temperature hydration of MOC by destabilizing the [Mg(H_2_O)_6_]^2+^ hydration shielding layer formed around dissolving MgO particles. Under normal conditions (left image), MgO dissolution releases Mg^2+^ ions that are surrounded by coordinated water molecules, forming a stable [Mg(H_2_O)_6_]^2+^ complex. This hydrated layer creates an electric double layer at the MgO surface, which limits further Mg^2+^ diffusion into the pore solution. When inorganic salts are introduced (right image), additional ions modify the interfacial electrochemical environment. These salts increase the Zeta potential of the MgO surface, enhancing electrostatic repulsion within the hydration shielding layer. Consequently, the concentration of hydrolyzed magnesium complexes rises, accelerating hydroxyl bridge formation and promoting the competitive reaction pathway toward the 5 phase [130].

According to the report, after being soaked for 28 days, the samples containing 30% fly ash maintained 80% of their strength. Furthermore, the water resistance of these samples was significantly better than that of the samples without fly ash [131]. By adding fly ash to MOC cement, the curing process will involve more amorphous phases [132], like *M*-*Cl*-*S*-*H* gel (gel composed of magnesium chloride, silicate, and hydrate) and *M*-*Cl*-*H* gel. By incorporating fly ash, the crystal phases are made more stable, which causes them to manifest as flocculation, the acicular phase 5. The addition of Silica Glass Powder (SGP) can result in a delay of the setting time of MOC cement, as well as an improvement in its water-resistant properties. However, this modification may also cause a slight reduction in the strength of the cement [94]. Forsterite formation will fill the pores in the cement, which will prevent phase 5 from decomposing and stop the creation of brucite [94]. The combination of MOC cement with incinerated sewage sludge ash (ISSA) results in the formation of an insoluble gel called *M*-*Cl*-*S*-*A*-*H*, which is able to shield phases 3 and 5 from hydrolysis [133]. To enhance the water resistance even more, it is possible to add a small quantity of calcium element to the slag. This will enable the strength of the material to be preserved more effectively [134]. Studies suggest that MOC cement’s water resistance can be improved by using inorganic elements such as rice husk ashes (RHAs) [135] and FeSO_4_ [136]. Adding inorganic fillers can have a negative impact on certain properties of MOC cement. For example, the addition of phosphoric acid [23] as a retarding agent can decrease the micro-compactness of MOC, resulting in weakened strength. In addition, it is important to be mindful of the quantity of filler being used. Using too much can cause the sample to slow down, crack, and display other imperfections.

### 6.2. Organic Additives

Typically, polymers exhibit favorable resistance to water or are hydrophobic. Polymer materials can be added to MOC cement as an organic additive to enhance the water-resistant characteristics of the resulting MOC cement products [137]. Typically, water-resistant modifiers that are organic are made up of water-soluble or emulsion-based polymers. These polymers can be mixed evenly with the MOC cement matrix, preventing clumping. The hydration process of MOC cement will remain unaffected by this type of modifier. Typically, it is applied on the external surface of crystal phases to obstruct the union of Cl ions and water. Additionally, it occupies the tiny pathways within the material to prevent water penetration and decrease the likelihood of scum buildup [138]. Wang et al. [139] discovered that the EVA emulsion is well-matched with MOC cement. This can enhance the connection between the glass fiber and the crystal, resulting in an improved ability to resist stress and prevent cracking.

A lone modifier has a restricted capacity to alter a substance, and it can also cause deterioration in other qualities. The combination of different modifiers can unexpectedly impact MOC cement. Xu et al. [135] combined a styrene/acrylic emulsion with calcium superphosphate to create a water-resistant composite material. The addition of the inorganic additives led to a 0.97 increase in the softening coefficient. In addition, at room temperature, the MOC cement bricks that were treated with this modifier did not experience scumming, warping, or contracting for a period of 360 days. A mixture containing stearic acid, styrene acrylic acids, and phosphate was developed [101]. This mixture acts as a compound modifier and is capable of reducing water absorption and preventing buckling deformation effectively. The issue of low water resistance was resolved with the assistance of phosphate ions. Further, the copolymer emulsion’s hydrophobic groups may enter the MOC cement’s pores and capillaries to some extent, and the groups will align on the crystal phases’ surface. By doing so, the structure became denser, and the quality of the pore surfaces was enhanced. The modifier has two primary objectives: the first one is to boost MOC cement’s compressive or flexural characteristics, while the second one is to enhance its resistance to water, which is also the subject of investigation. The aim is to avoid the crystal phases from coming into contact with water, which could lead to hydrolysis, by either creating a gel phase that is resistant to water or blocking the micropores. Structural design can be utilized to achieve hydrophobicity in addition to the use of additives. Gao et al. [140] made MOC superhydrophobic through nano-casting technology, resulting in a self-cleaning surface function.

### 6.3. Fiber Reinforcement

**Figure 16:**
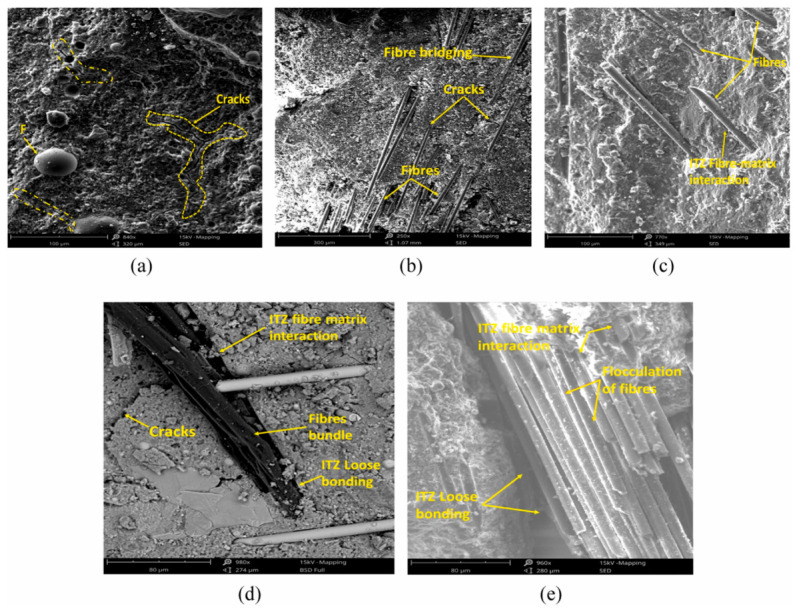
Fiber bridging effect in MOC specimens containing PE and basalt fiber [87].

Fibers are frequently utilized in engineering applications to enhance the durability of cement, as it is a brittle substance. MOC cement contains a significant quantity of chlorine ions that possess a potent ability to corrode metals [141]. As a result, utilizing steel as a means to improve MOC cement proves to be challenging [142,143], while plant fiber, glass fiber, and polymer fiber may all be used as reinforcing materials [71,144]. By adding glass fiber and a water-resistant modifier to MOC cement, it was possible improve its water resistance [145]. Wood–MOC composites were strengthened by Zhou et al. [146] using glass fibers and polyvinyl acetate (PVA). By using extrusion molding, the fibers were arranged in a specific direction within the cement mixture, resulting in an enhancement of the mechanical characteristics of the composite material. He et al. [102] suggest that waste wood fiber has been incorporated into MOC cement in order to create wood–plastic composite materials that possess desirable qualities such as high heat retention, noise reduction, and flexural strength. Composite materials offer the benefit of being eco-friendly and can serve as effective materials for both thermal insulation and soundproofing. Wang et al. [147] created materials using straw or MOC that were lightweight, but they discovered difficulties with the connection between the fiber and the MOC cement. Because the surface of straw has a smooth and waxy layer, it creates feeble interface layers between the straw and the matrix. As a result, it becomes challenging to establish a strong bond between the straw and the matrix. The full potential of the crack resistance effect of plant fiber was not utilized when the microcracks formed and spread to the outer edges of the straw, resulting in the complete removal of the straw from the section. Figure 16 highlights the fiber bridging mechanism, where well-dispersed PE/BF fibers span across microcracks and anchor into the surrounding MOC matrix through a dense interfacial transition zone (ITZ). These fibers act as stress-transfer elements, delaying crack propagation by converting localized tensile stresses into distributed pull-out and debonding resistance. In mixes with optimal dispersion, fibers are uniformly embedded within the compact phase 5 needle network, forming a stable three-dimensional bridging system that enhances crack arrest, load redistribution, and post-peak ductility [87].

### 6.4. Mechanical Performance of Modified MOC System

Across recent studies, the mechanical performance of modified MOC is governed by how additives preserve or promote interlocking phase 5 hydrate networks (refine pore structure and ITZ) and introduce crack-bridging or film-forming mechanisms that improve post-peak response without sacrificing early strength [103,148,149]. Replacing a small fraction of reactive MgO with fly ash (FA) and/or metakaolin (MK) can reduce cost and tune kinetics, but excessive replacement tends to dilute the reactive fraction and reduce compactness/strength. Gong et al. [148] showed that FA typically retards setting and slows hydration, whereas MK can accelerate the early reaction; importantly, FA/MK do not fundamentally change the hydrate types but influence the morphology of phase 5 and the packing density of the matrix. Moving from paste/mortar to engineered composites, GGBFS and MK can be used to enhance mechanical capacity when combined with fiber reinforcement. Ahmad et al. [149] reported rapid early strength development (1-day compressive strength ≈ 69–84% of 28-day values) and identified an optimum hybrid system (30% GGBFS with hybrid PE/BF fibers), achieving 73.9 MPa compressive strength together with high tensile capacity (8.52 MPa) and strain capacity (2.22%). This indicates that, in well-designed mixes, SCMs can support high macro-strength while also enabling ductility when the fiber–matrix bond and dispersion are controlled [149].

Although phosphate modifiers are often discussed for water resistance, their mechanical significance is that they allow strength to be retained in aggressive moisture exposure and can shift the flexural–compressive balance. In a direct comparison of sodium monofluorophosphate (MFP), phosphoric acid, and soluble phosphate (KH_2_PO_4_), all three increase flexural strength, slightly reduce compressive strength, and markedly improve strength retention [99]. Mechanistically, these additives change phase 5 growth direction/morphology and the micropore system, which is highly relevant for mechanical reliability because MOC components often fail by microcrack growth and intergranular debonding once the structure hardens. A key strategy to raise mechanical robustness while improving durability is to refine porosity and create secondary binding gels. Guo et al. [103] demonstrate that an MOC blend containing 15% silica fume + 15% fly ash can retain compressive strength at ~100% after 28-day water immersion (and ~95% after 56 days), attributing this to pore structure optimization and formation of Mg–Cl–Si–H gel, alongside a stabilized phase assemblage that relieves internal stresses [103]. From a mechanical standpoint, this is important because it couples high compressive capacity with microstructural stability, limiting damage accumulation in service.

Polymer modification strengthens MOC performance primarily by forming continuous films and grid-like networks that restrain hydrate decomposition and microcrack propagation. For foamed MOC, EVA powder slows hydration and inhibits Mg(OH)_2_ formation; when EVA exceeds ~9%, the film becomes continuous and improves pore structure while stabilizing phase 5 in water, supporting better compressive performance for lightweight systems [97]. At the paste level, epoxy and polyurethane modifications are explicitly aimed at improving water submergence performance while preserving mechanical properties over immersion periods [150]. In high-ductility composites, the strongest mechanical gains occur when SCM densification is paired with fiber bridging: Ahmad et al. showed tensile strain-hardening in all mixes and linked superior strength/strain capacity to improved fiber dispersion and a dense ITZ [149].

Industrial by-products can improve mechanical performance by promoting secondary gel phases and refining pores. Feng et al. [85] reported that incorporating 20 wt% biomass fly ash optimizes hydration and compressive strength at 7 days, with secondary M-S-H gel formation and densification identified as key. A complementary pathway is carbonation-enabled strengthening: using solid-waste-derived MgO sources, an optimized MOC reaches 91.17 MPa compressive strength at 28 days and develops hydrated magnesium carbonate phases that fill pores and can enhance strength and stability [151].

## 7. Discussion

The literature on MOC has expanded rapidly and now spans fundamental chemistry, composite design, durability enhancement, and emerging multifunctional applications. However, a recurring challenge is that many findings are difficult to compare directly because MOC performance is strongly conditioned by mixture design parameters (e.g., MgO/MgCl_2_/H_2_O ratios), MgO reactivity, impurity chemistry, curing temperature/humidity, and even the operational definition of “water resistance” [19,20,22]. A consistent trend across the MOC literature is that nominally similar additive strategies can yield different outcomes when applied under different mixture ratios and curing regimes. The reason is that the mechanism, including hydration product assemblage (particularly the formation, morphology, and stability of MOC phases), is controlled by the chemical environment and kinetics, which are in turn shaped by MgO reactivity and curing conditions [19,20,22]. Studies that omit critical descriptors (reactivity indices for MgO, MgCl_2_ concentration, and curing humidity/temperature) effectively prevent reproducible comparison. This review therefore interprets reported improvements (strength, softening coefficient, and strength retention under immersion) as conditional improvements unless the governing variables are clearly reported and comparable.

Table 2 presents a comparative overview of experimental investigations on magnesium oxychloride cement (MOC), including raw material ratios, curing conditions, mechanical properties, and durability indicators. Across the literature, the stability and morphology of phase 5 emerge as the dominant factors controlling both strength development and water resistance. The results indicate that water-induced degradation is primarily associated with the hydrolysis of phase 5 into Mg(OH)_2_ and the consequent increase in porosity. Strategies such as phosphate modification, SCM incorporation, carbonation curing, polymer surface treatment, and process optimization have been shown to mitigate this degradation through pore refinement, reduction in free Mg^2+^ concentration, protective precipitation layers, or formation of hydrated magnesium carbonates (HMCs). The compiled data underscore that optimizing MOC requires a balance between phase assemblage control and microstructural densification rather than strength enhancement alone.

Water resistance remains the most widely recognized barrier to broader deployment, and recent reviews dedicate substantial effort to this theme [22,41,42]. Across the evidence base, three strategy families appear repeatedly: (i) phosphate-based or acid-based chemical modification; (ii) SCMs that refine pore structure and alter transport; and (iii) organic/polymeric networks that introduce hydrophobicity and crack-bridging. From a comparative standpoint, soluble phosphates and phosphoric acid-type modifiers are among the most mechanistically grounded approaches because they can change dissolution–precipitation pathways and promote the formation of less water-susceptible products and denser microstructures. The mechanistic basis for phosphate-driven improvement is discussed in foundational work [117] and further supported by later experimental studies, including phosphoric acid modification used to improve performance for biomaterial-oriented MOC systems [23]. However, an important trade-off emerges: phosphate/acid systems can also retard setting or reduce early strength if the dosage is not optimized, making “maximum water resistance” a multi-objective problem rather than a single-variable target [22,42].

SCM incorporation (e.g., fly ash and other reactive mineral additions) is frequently reported to improve water resistance through micro-filling, reduced connectivity of capillary pores, and altered interfacial microstructure. Crucially, not all SCMs behave equivalently; comparative evidence indicates that improvement depends on SCM chemistry and its interaction with the MOC hydration environment [26]. The trade-off here is “dilution vs. refinement”: higher replacement may reduce the amount of reactive MOC-forming constituents, potentially lowering early-age strength, even while improving immersion resistance. This explains why some studies reported optimal SCM dosages rather than monotonic improvement [22,26]. Polymeric modifiers and hybrid organic–inorganic concepts offer a different pathway; rather than primarily changing hydration products, they modify transport (reduced water ingress) and fracture behavior (bridging/ductility), which can indirectly enhance water resistance by limiting microcrack formation and connectivity. Recent reviews highlight the promise of these routes but also note the need for scale-up and systematic validation for construction products [41,42]. A key limitation is that polymeric modification may increase cost and complicate processing, so performance gain must be evaluated alongside practical manufacturing considerations.

Compared with OPC systems, MOC appears particularly sensitive to the curing regime. Evidence shows that curing conditions measurably influence mechanical performance and moisture resistance [22,74]. Moreover, CO_2_ curing has emerged as a potentially valuable lever to improve performance, but the literature is still not mature enough to establish universally applicable design rules [41]. The key comparative implication is that “additive effectiveness” should not be interpreted independently of curing: an additive that performs strongly under controlled laboratory curing may not retain the same benefit under variable field curing unless the curing pathway is part of the design specification.

Even if immersion resistance is improved, broader implementation requires addressing durability and constructability constraints, such as reinforcement compatibility and corrosion risk in chloride-bearing environments. Since MOC systems rely on MgCl_2_, the risk profile for embedded steel differs from OPC and needs explicit treatment. Corrosion-related studies on steel in MOC concrete and anticorrosion strategies indicate that reinforcement durability is a non-trivial barrier and must be treated as a first-order design consideration rather than an afterthought [35]. This observation partially explains why many practical MOC applications emphasize boards, panels, flooring, and composites where steel reinforcement demands are reduced [22,41]. A further durability-related comparability issue is that studies use different exposure protocols (immersion durations, drying–wetting cycles, and temperature conditioning), leading to different degradation pathways. This reinforces the need for standardized durability testing frameworks tailored to MOC systems, as also implied by the evidence synthesis limitations identified in prior reviews [22,42].

MOC is frequently positioned as a lower-carbon alternative binder, but sustainability claims must be supported by consistent boundary conditions (raw material sourcing, calcination energy, transport, and end-of-life). Comparative environmental assessments indicate that MOC-based solutions can offer environmental advantages in specific construction systems (e.g., wall assemblies), but outcomes depend strongly on supply chain and system-level design choices [59]. Meanwhile, studies on CO_2_ uptake and carbonation-related behavior suggest that MOC-based materials may offer measurable CO_2_ capture potential under certain conditions [34,151].

## 8. Conclusions

This review critically examined the current state of research on MOC, integrating findings from fundamental hydration studies, microstructural investigations, durability enhancement strategies, and application-oriented developments. By synthesizing evidence across mixture design, curing regimes, additive modifications, and performance evaluation, the review provides a consolidated understanding of the progress achieved to date.

1. MOC demonstrates significant potential as a high-early-strength, rapid-setting binder suitable for boards, panels, and composite construction products; however, its long-term durability, particularly moisture and immersion resistance, remains the primary constraint limiting widespread structural application.
2. MOC performance is highly system-dependent. Variations in MgO reactivity, mixture ratios, MgCl_2_ concentration, and curing regimes substantially influence hydration kinetics, phase assemblage, and durability outcomes. Many findings in the literature can therefore be attributed to differences in process control rather than fundamental material inconsistencies, highlighting the need for standardized reporting and testing protocols.
3. Water-resistance enhancement strategies, including chemical modification (e.g., phosphate-based approaches), supplementary cementitious material incorporation, and polymeric or hybrid modifications can significantly improve performance. However, these strategies involve trade-offs in workability, setting time, strength development, long-term stability, and cost. Optimal mix design should thus be approached as a multi-objective optimization problem rather than a single-performance improvement.
4. Translation to structural applications requires explicit attention to reinforcement compatibility and corrosion risks associated with chloride-bearing systems, as well as broader durability considerations beyond short-term immersion testing. Without addressing these aspects, large-scale adoption in reinforced concrete systems will remain limited.
5. MOC exhibits promising sustainability potential, particularly in terms of lower calcination temperature and possible CO_2_ uptake; its environmental performance is application-dependent and requires harmonized lifecycle assessment frameworks and system-level validation.

Based on the evidence synthesized and the comparative analysis developed in this review, the most impactful future directions for MOC research include: (i) standardized reporting and testing frameworks that control for MgO reactivity, mixture ratios, curing regime, and water-resistance assessment protocols; (ii) mechanistic studies linking additive chemistry to phase stability under coupled moisture–temperature exposure, moving beyond single-condition immersion testing; (iii) scale-up studies that validate lab-scale improvements under realistic curing and service conditions; (iv) explicit durability design strategies for reinforcement compatibility and corrosion mitigation in chloride-bearing environments; and (v) harmonized LCA studies that quantify when and where MOC systems deliver net environmental benefit, including the role of CO_2_ curing and CO_2_ uptake in whole-life performance.

Future research on MOC should focus on bridging the gap between promising laboratory-scale performance and reliable field-level applications. Addressing the long-standing challenge of moisture sensitivity remains critical, requiring the development of robust, scalable, and cost-effective modification strategies that enhance durability without compromising mechanical performance. Given the strong system dependency of MOC, there is a dire need for standardized mix design protocols, testing methodologies, and reporting frameworks to ensure reproducibility and comparability across studies. Advancing MOC toward structural applications further requires comprehensive investigations into reinforcement compatibility, chloride-induced corrosion risks, and long-term durability under realistic service conditions. Additionally, mix design optimization should adopt a multi-objective approach that balances strength, durability, workability, and economic feasibility. Finally, to fully realize its sustainability potential, future work must incorporate harmonized life-cycle assessment and system-level validation to quantify environmental benefits across different circular-economy applications [165,166].

## Figures and Tables

**Table 1:** Stages of hydrated MOC cement.

Phase	Composition	Formed Temperature	Structure
**3-1-8 (phase 3)**	3Mg ${\left(OH\right)}_{2}\cdot MgCl_{2}\cdot 8H_{2}O$	Approximately 10–55 °C [69]	Figure 8a shows chains of octahedra consisting of two Mg ${\left(OH\right)}_{4}{\left(OH_{2}\right)}_{2}$ units with $Cl^{-}\;$ and $H_{2}O$ molecules inserted between them [80].
**5-1-8 (phase 5)**	5Mg ${\left(OH\right)}_{2}\cdot MgCl_{2}\cdot 8H_{2}O$	Approximately 10–30 °C [68,70]	The structure consists of three chains that are made up of one Mg ${\left(OH\right)}_{6}$ octahedron and two Mg ${\left(OH\right)}_{4}{\left(OH_{2}\right)}_{2}$ octahedrons [80]. These chains contain $Cl^{-}$ and $H_{2}O$ in a disordered manner as shown in Figure 8b.
**2-1-2**	2Mg ${\left(OH\right)}_{2}\cdot MgCl_{2}\cdot 2H_{2}O$	Beyond 100 °C [77]	The structure is composed of an endless series of triple chains that are connected by edges, and each chain is made up of bent $\;MgO_{6}\;$ octahedra [75].
**2-1-4**	2Mg ${\left(OH\right)}_{2}\cdot MgCl_{2}\cdot 4H_{2}O$	Beyond 100 °C [77,78]	The passage is referring to a structure composed of parallel components, which are connected by zigzag chains of disordered chloride ions $\left(Cl^{-}\right)$ and water molecules [77].
**9-1-4**	9Mg ${\left(OH\right)}_{2}\cdot MgCl_{2}\cdot 4H_{2}O$	Beyond 100 °C [77,78]	The text describes a structure composed of an endless series of three chains of $MgO_{6}$ octahedra connected by two chains [78].
**Chlorocarbonate**	$Mg_{2}(OH)$ $Cl\cdot CO_{3}\cdot 2H_{2}O$	Room temperature [80]	Figure 8c shows a ring with 15 members that is distorted around an octahedral structure containing magnesium [80].

**Table 2:** Mix design parameters: fresh, mechanical, and durability properties of MOC.

Ref	Binder		Curing Regime	Fresh Properties	Mechanical Properties	Durability Assessment	Remarks
	Type (wt%)	Molar Ratio					
Dai et al. [152]	MOC (100) + SA (0.2–0.6% of MgO) + PAA+ CA	5:1:13 (MgO: MgCl_2_: H_2_O)	25 °C, 60% RH	- Viscosity: 2589 SA ↑ to 9891; PAA/SA ↓ to 1746 mPa·s	- f`c: 52.5 MPa (28d) - PAA/SA ↑ long-term - Dry/wet shear: 3.13/2.48 MPa	- SC: 0.31 (MOC)→ 0.97 (PAA/SA2) - Porosity ↓ 31.1%→17.5%	- ↑ P5 (62.6%→67%); ↓ MgCO_3_ - Mg(OH)_2_ formation reduced - LCA: CO_2_ ≈ 20% of PF
Cui et al. [89]	MOC (100) + FC (CaO: 0–10% of MgO)	3:1:11 (M3); 5:1:13 (M5)	25 °C 75% RH	- Flow: ↑ then ↓ with FC. - Setting time: ↑ (≤4% M3/≤2% M5), then ↓.	- 28d f`c ↓ with FC; 5.6%↓ for M3 and 6.9% ↓for M5.	—	- Phase transformation: M3→ P3→P5 when FC >4%. - M5 → P5→Mg(OH)_2_ when FC >2%. - Permissible FC: <4% (M3), <2% (M5).
Liu et al. [153]	MOC (100) + RM, Fa, GZ, FS, KHP (all 10% MgO)	8:1:14	Air: 50 °C and simulated well: 50 °C, 21 MPa water	—	- f`c ↓ 77.23% (1d→14d) for water cured. - f`c ↑ 43.23% (1d→28d) for air cured.	- Permeability: MOC–water ↑ by 441.84%. - 28d permeability ↓ 75.98% vs. control.	- Under hydrothermal: P5 → Mg(OH)_2_ (hydrolysis). - ↑ porosity and ↓ Mg(OH)_2_.
Aiken et al. [154]	MOC (100) + FA, Slag, MK-A, MK-B (10–30% MgO repl.)	9.6:1:15 reduced to 6.8:1:15	20 ± 2 °C, 50 ± 5% RH	- FA/Slag: Flow ↑. - MK-A: Flow ↓. - Setting time ↑.	- 28d f`c ≈ 100 MPa at 30% repl. - early CS ↓ (all SCMs).	- Wn: Control = 0.31, FA ≈ 0.48–0.55, Slag ≈ 0.45, MK-A = 0.77, MK-B = 0.64.	- P5 ~53–61%. - Improved water resistance linked to ↓ MgO → ↓ brucite formation. - MK ↓ porosity to 1.77–1.96%
Lv et al. [155]	- MgO-a - MgO-b - MgO-c - MgO-d	7:1:15	20 ± 3 °C RH 50% for 3–28 d	—	**28d f`c:** - MOC-a = 75.4 MPa. - MOC-b = 69.6 MPa. - MOC-c = 50.1 MPa. - MOC-d: failed (flash set)	- Porosity (MIP): MOC-c lowest; MOC-b ≈ MOC-a	- MgO (a,b,c) activity = 74.1–81.7% (> raw 53.1%). - MgO-d activity = 14.5%. - Large dense particles in MgO-b.
He et al. [26]	MOC (100) + PFA (30% MgO repl.) + ISSA (30% MgO repl.)	MgO/MgCl_2_ = 9; H2O/MgCl_2_ = 10	25 °C, RH 50%; 14d air → 28d immersion	—	- 14d f`c: control= 165 MPa, PFA = 112 MPa, ISSA = 150 MPa. - 28d immersion SR: Ctrl = 10%; PFA = 73%; ISSA = 87%	- Expansion: Control = 1.8%; PFA = 0.6%; ISSA = 0.25%	- Amorphous Mg–Al–Si gel formed. - P5 morphology changed (needle → lath/fibroid). - ISSA produced higher amorphous content (~76% vs. 24.7% PFA).
Xie et al. [86]	MOC (100%) + FA (10%, 30%, 50% MgO replacement)	7:1:15	A: 60 ± 5% RH, 20 °C. C: 20% CO_2_, 60 ± 5% RH, 20 °C, 12 h → air	- Flow ↑ 1.3–11.4%. - Initial setting time ↑ 8.8–83.2%	- FA ↓ early f`c (−32–66%). - C curing ↑ f`c 0.4–26.9%. - 90d f`c up to 101 MPa (C)	- Softening coefficient: 0.025 (MOC-0,A) → 0.832 (MOC-50,C). - Water absorption ↓ 9.3–60.5% (C). - Porosity ↓ 2.6–29.1% (C).	- FA forms M-S-H/M-Cl-S-H gel. - C forms HMC (MgCO_3_, Mg_2_CO_3_(OH)Cl·3H_2_O). - CO_2_ sequestration ↑ 7.1–12.9%
Li et al. [156]	MOC (100) + HPDMS (0–10 wt%)	MgO 250 g; MgCl_2_·6H_2_O 103.7 g; H_2_O 67.6 g	25 °C 60% RH, 28 d air	—	- f`c↓ with HPDMS: 105.5 MPa (0%) → 71.3 MPa (10%)	- Softening coefficient: 27.3% (0%) → ~100% (≥6% HPDMS). - Water absorption↓ to 0.25% (10%)	- Superhydrophobic (CA > 150°, SA < 10° at ≥6%). - Dual-level fractal structure. - Anti-icing.
Feng et al. [85]	MOC (100) with RM, FA, MK, slag (5–30% MgO repl.)	MgO/MgCl_2_ = 5; H_2_O/MgCl_2_ = 15	20 °C, RH 60% for 1–28 d	- Calorimetry: SCM ↓ heat flow and Q (24 h) (to ~76% at 30%)	28d f`c (opt.): - FA5 = 54.4 MPa - S20 = 43.9 MPA - RM20 = 39 MPa - MK20 = 41 MPa	Softening coefficient (7d imm.): - RM20 = 11.1% - FA30 = 45.2% - MK30 = 76.8% (best) - Slag ≤ 16%	- P5 dominant. - Amorphous M-S-H/Mg-Cl-Si-H gel (SEM/EDS). - Excessive SCM ↓ MgO → weaker P5 framework.
Cao et al. [157]	MOC (100) with HBSA (0–30%)	MgO/MgCl_2_ constant	28 d @20 °C, erosion: salt brine, freeze–thaw (FT), salt-freezing (SF) (up to 60 cycles)	—	- 10% HBSA: W_3_ ↑ 21.24% (brine); ↑23.48% (FT); ↑18.91% (SF) vs. 0%	- FT most severe, SF intermediate, brine least. - 10% HBSA ↓ harmful pores by 18.89% (FT). - Open porosity ↓5.04% (FT)	- P5 dominant. - Erosion → 5-phase ↓, Mg(OH)_2_ ↑. - HBSA → M-S-H gel formation. - SF damage due to salt crystallization + freezing expansion.
Wang et al. [129]	MOC (100), FA (20, 30, 40,60% binder), PE fiber (2 vol%)	MgO/MgCl_2_ = 6	28 d air + 28 d water soaking	- Flow ↑ at 30% FA (max 180 mm)	- Tensile strain 5–8%. - ft >7 MPa. - f`c up to 35.8 MPa (air). - Post-peak ductile	- SR ≈ 0.80–0.93. - Tensile ductility maintained. - Crack width <120 μm	- P5 ↓ after water; brucite ↑. - FA → refined matrix + defect control. - M-S-H gel restrains phase 5 decomposition
Li et al. [151]	MOC (100), CCR-MgO (10–30% MgO repl.)	W/C = 0.60, MgCl_2_ 25°Bé	25 ± 3 °C; CO_2_ = 20%; RH = 40%	—	- 28d f`c: = 91.17 MPa (max), natural 82.48 MPa	- Water resistance ↑ after carbonation. - Retention = 60.73% (vs. 25.25% natural).	- HMC ↑ (8.06%). - Chlorartinite → hydromagnesite. - Capillary pores ↑ → CO_2_ diffusion ↑.
Power et al. [158]	MOC (100), brucite (6–28 wt%)	—	Ambient aging (0–15 yrs), accel.: 10% and 100% CO_2_	—	—	- Passive carbonation rate ≈ 0.07 kg CO_2_/m^2^·yr (≈9.8 kg CO_2_/t·yr). - 20–40% CO_2_ offset over 15 yrs. - Accelerated carbonation ↑400–1600×.	- Secondary HMC (hydromagnesite, chlorartinite) from phase 5 + brucite. - Carbonation in interfacial H_2_O layers. - Pore infilling + surface passivation.
Meng et al. [159]	MOC (100)	_	Carbonation curing: 5–100% CO_2_, 30–120 °C; RH 55–98%	_	- f`c: 60–150 MPa (28 days)	- CO_2_ sequestration: MOC ≈ 1 kg CO_2_/m^2^ (15 yrs passive)	- Optimal CO_2_ ≈ 5–10% and RH ≈ 78%. - MOC forms chlorartinite. - Carbonation improves water resistance.
Zheng et al. [160]	MOC (100) +FA (0–60% binder)	a-MgO/MgCl_2_ = 7:1; W/C = 0.50	20 ± 3 °C, RH ≈ 50%; 3–28 d	- Flow ↑ (206→297 mm at 60% FA). - Setting time ↑ linearly	- 28d f`c ↓ with FA: −14% (10%), −28% (20%), −38% (30%), −49% (40%), −61% (50%).	- Porosity ↑ (20% FA: +80% total porosity). - Mean pore size ↑.	- P5 dominant; FA ↓ P5 content. - FA improves workability. - Hydration heat ↓. - Induction period ↑.
Huang et al. [161]	PG+ MOC, NaHCO_3_ (0–2% PG)	MgO/MgCl_2_ = 1:1–7:1 (opt. 3:1)	20 ± 1 °C, RH > 95%, 7–90 d; water imm.	_	- Max UCS = 6.25 MPa (7d, 3:1, 0.5% NaHCO_3_)	- Softening coefficient = 74.28% (0.5% NaHCO_3_). - Water absorption ↑ with NaHCO_3_.	- Phase 5 + nesquehonite. - NaHCO_3_ ↑ hydration rate. - Carbonation → columnar nesquehonite.
Yuan et al. [81]	MOC (100)	H_2_O/MgCl_2_ = 13–19; MgO/MgCl_2_ ≈ 6.2–8.7	20 °C; early-age (0–240 min)	- Yield stress ↓ with ↑H2O/MgCl_2_ - Plastic viscosity ↓. - Shear-thinning ↑.	_	_	- P5 formation accelerates rheology evolution. - ↑H_2_O/MgCl_2_ delays setting. - Ionic strength and pH control ζ-potential.
Pu et al. [162]	MOC (100%); NaH_2_PO_4_ (0, 1, 2, 4%)	MgO/MgCl_2_ = 7; H_2_O/MgCl_2_ = 12	28 d air; + 7 d and 28 d water imm.	—	- 28d f`c: ↓ with ↑NaH_2_PO_4_(−5%, −9%, −28%); Opt. = 2%	- Softening coefficient: P-2 ≈ 0.91; P-1 ≈ 0.86; P-4 ≈ 0.78; P-0 ≈ 0.13	- Phosphate ↑ phase 5 (air). - Excess PO_4_^3−^ damages phase 5. - P-2 densest pore structure.
Li et al. [163]	MOC (100) + sawdust (20% of active MgO) + NaH_2_PO_4_ (0.5–2%)	Opt.: MgO/MgCl_2_ = 11; MgCl_2_ sol. = 25%	28 d air; + 28 d water imm	—	- 28d f`c (opt.) ≈ 75.96 MPa. - 28d flexural strength (opt.) ≈ 21.03 MPa	- Opt. softening coefficient ≈ 0.919. - Water absorption ↓ to ≈ 4.99%	- ↑ MgCl_2_ conc. → ↑ P5; NaH_2_PO_4_↑ WR but ↓ strength at high dosage. - Excess MgCl_2_ → Mg(OH)_2_ formation after imm
Wang et al. [164]	MOC (100)	MgO: MgCl_2_: H_2_O = 7.28:1:14.09	Opt. curing T = 42.14 °C; RH ≈ 60%; 7d air + 7d imm	—	- 7d f`c (opt.) = 90.87 MPa	- Softening coefficient (opt.) = 0.51	- P5 dominant strength phase. - Excess MgO → Mg(OH)_2_ after imm. - High T (>50 °C) ↑ pores. - Opt. T (≈40–45 °C) → denser microstructure.

SA: sodium alginate; PAA: polyacrylic aci;, f`c: compressive strength; SC: softening coefficient; P3/5: phase 3/5; LCA: lifecycle assessment; FC: free CaO; RM: red mud; FA: fly ash; GZ: green zeolite; FS: ferrous sulfate heptahydrate; KHP: potassium dihydrogen phosphate; (C): carbonated; MK: metakaolin; Wn: strength retention coefficient; MgO-a: waste MOC block; MgO-b: crushed waste MOC; MgO-c: water-soaked waste MOC; MgO-d: direct bischofite calcination; PFA: pulverized fuel ash; ISSA: incinerated sewage sludge ash; SR: strength retention; HPDMS: hydroxyl-terminated polyimethylsiloxane; CA: contact angle; SA: sliding angle; HBSA: highland barley straw ash; ft: tensile strength; W/C: water/cement ratio; HMC: hydrated magnesium carbonate; CCR-MgO: MgO from calcium carbide; PG: phosphogypsum; and UCS: unconfined compression strength.

## Data Availability

No new data were created or analyzed in this study. Data sharing is not applicable to this article.
